# Chemical Constituents and Their Production in Mexican Oaks (*Q. Rugosa, Q. Glabrescens* and *Q. Obtusata*)

**DOI:** 10.3390/plants11192610

**Published:** 2022-10-04

**Authors:** Elgar Castillo-Mendoza, Alejandro Zamilpa, Manasés González-Cortazar, Ever A. Ble-González, Efraín Tovar-Sánchez

**Affiliations:** 1 Centro de Investigación en Biodiversidad y Conservación, Universidad Autónoma del Estado de Morelos, Av. Universidad 1001, Col. Chamilpa, Cuernavaca 62209, Morelos, Mexico; 2 Centro de Investigación Biomédica del Sur, Instituto Mexicano del Seguro Social, Argentina No. 1, Col. Centro, Xochitepec 62790, Morelos, Mexico; 3 División Académica de Ciencias Básicas, Universidad Juárez Autónoma de Tabasco, Carretera Cunduacán-Jalpa Km. 0.5, Cunduacán 86690, Tabasco, Mexico

**Keywords:** HPLC, phenolic compounds, TLC, terpenoids, specialized metabolites

## Abstract

Mexico is considered one of the main regions of diversification of the genus *Quercus* (oaks). Oak species are one of the most important tree groups, particularly in temperate forests, due to its diversity and abundance. Some studies have shown that oak contains specialized metabolites with medicinal importance. In this work, the acetonic extract from leaves of three Mexican oaks (*Quercus rugosa*, *Q. glabrescens,* and *Q. obtusata*) was separated using thin-layer chromatography and column chromatography. Chemical identification of the major compounds was determined using high-performance liquid chromatography and nuclear magnetic resonance. Nineteen compounds were identified, three belonging to the terpenoid family (ursolic acid, β−amyrin, and β−sitosterol) and 16 from the phenolic family. Of the isolated compounds, seven are new reports for oak species (scopoletin, ursolic acid, β-amyrin, luteolin−7−O−glucoside, kaempferol−3−O−sophoroside, kaempferol−3−O−glucoside, and kaempferol−3−O−sambubioside). More compounds were identified in *Q. rugosa* followed by *Q. glabrescens* and then *Q. obtusata*. The characterization of specialized metabolites in oak species is relevant, from both phytocentric and anthropocentric perspectives.

## 1. Introduction

Plants naturally produce many specialized metabolites which, although not related to essential functions, are involved in processes that impact survival and reproduction. For example, specialized metabolites (sm) can lead to the following functions in the plants that produce them: protection from UV light, pigmentation, aromatic profile, plant hormones, co-factors in enzymatic processes (catalysts), allelopathic compounds, and defense against herbivory. They also influence other interactions that plants establish with diverse biological groups [1,2,3]. Specialized metabolites can be classified into four major groups: alkaloids, phenolic compounds, sulfur-containing compounds, and terpenoids [4].

Oaks (Fagaceae: *Quercus* L.) possibly originated in the Paleogene and spread through various environments during the late Cenozoic [5]. At present, they are the most important tree and shrub groups in temperate forests and are widely distributed globally [6]. *Quercus* is represented by approximately 500 species, which are found on four continents: Europe, Asia, North Africa, and North, Central, and South America [6,7,8]. Mexico comprises 30.3% of this diversity (161 species) and is considered one of the main regions of diversification of the genus [9]. In addition, it is estimated that oak forests occupy 7.5% of the national territory [10] and are particularly dominant in the Trans-Mexican Volcanic Belt (TMVB). Recently, it has been suggested that the diversification of this plant group in Mexico is related to the geological heterogeneity, climatic gradients, availability of habitats that promote oak populations, and the high genetic variability of the genus [9,11,12]. Finally, oaks interact with diverse biological groups (plants, epiphytes, arthropods, birds, small mammals, etc.), fulfilling diverse ecological functions. For example, they help to regulate climate, contribute biomass to nutrient cycling, participate in water balance, and are important from an economic point of view for human populations (providing food, fuel, construction materials, etc.) Furthermore, its chemical compounds, mainly phenolic compounds, are widely used in the context of the production of wine and whiskey. The leaves of some species are used for the cultivation of the silk moth, and the decomposing wood is used for the cultivation of fungi of economic importance, among which truffles stand out [7,13,14,15,16].

Despite the extensive use of some oak species such as timber, for example, in the wine industry, or as forage for animals (fowl and pigs) [17,18,19], there are few studies that characterize their chemical profile. In general, these works have documented the presence (qualitative) of various sm at the group or family level, specifically, tannins, phenols, flavonoids, aliphatic compounds, steroids, glucosides, hydrocarbons, and triterpenes [20,21,22,23]. In Mexico, there have been five studies documenting the presence of ellagitannins, phenolic compounds, flavonoid glucosides, and tannins in 16 oak species [24,25,26,27]. *Q. rugosa* is the only species reported in more than one study [24,27].

*Quercus rugosa*, *Q. glabrescens*, and *Q. obtusata* are three species of white oaks (section: *Quercus*) widely found in Mexico, mainly in the TMVB, over a broad altitudinal gradient (between 620 and 3300 m), and phylogenetically closely related [12], to high genetic diversity and reports of interspecific hybridization [27,28]. Given that Mexico is one of the main regions of diversification of the *Quercus*, but with limited knowledge of the diversity of sm that this genus may present, the analysis of the metabolic profiles presented by oaks is an important task in view of the ecological and economic implications that those profiles may have, as well as for their potential application in human health. For these reasons, the main aims of this study were to (1) determine the metabolic profile (majority compounds) of three species of white oaks (*Q. rugosa*, *Q. glabrescens,* and *Q. obtusata*) and to (2) find specific chemical markers for each study species.

## 2. Results

### 2.1. Yield of Acetone Extracts

Maceration of the aerial parts of the *Q. rugosa*, *Q. obtusata,* and *Q. glabrescens* (Qr, Qo, and Qg, respectively) species provided the following yields: Qr (eaQr, 12.46 g, 4.15%), Qo (eaQo, 16.83 g, 5.61%), and Qg (eaQg, 20.65 g, 6.88%). The three extracts were evaluated and compared with each other for their individual characterization, and the search for species-specific chemical markers (Table 1).

### 2.2. TLC, HPLC, and NMR Analysis of Extract and Fractions

**Figure 1:**
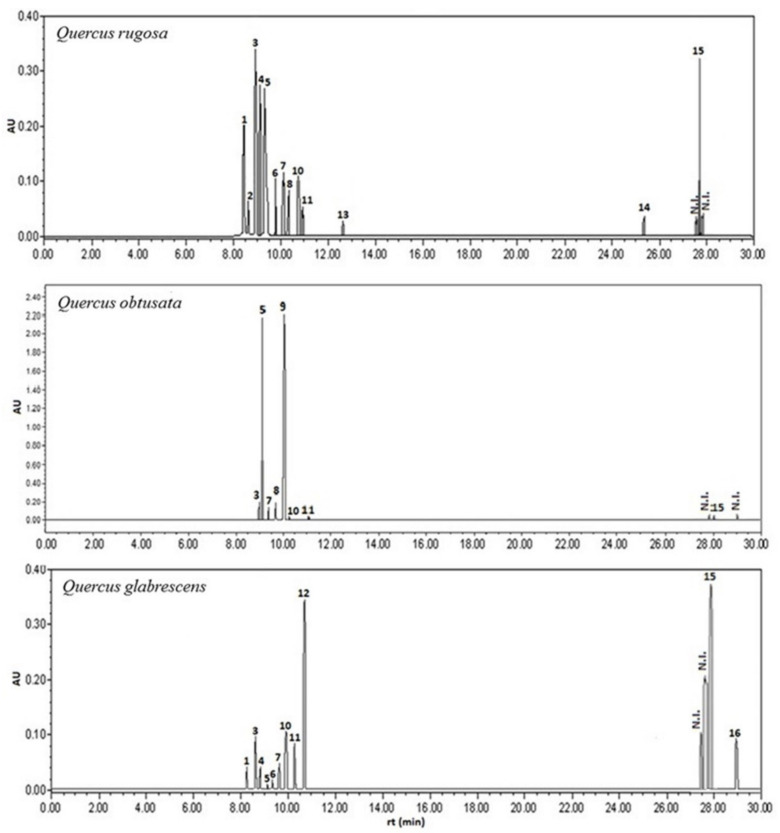
HPLC chromatogram of the phenolic compounds (**1**–**16**) in the three species of white oak (*Q. rugosa*, *Q. obtusata,* and *Q. glabrescens*). Wavelength of the chromatograms made at 320 nm. N.I. = not identified.

Analysis of acetonic extract by TLC, HPLC, and NMR allowed the identification of 19 compounds. Each compound had a unique retention time and a UV spectrum. HPLC analysis of the acetone extracts (λmax = 280, 320 and 350 nm) showed the presence of 16 polyphenolic metabolites (**1–16**, see Figure 1), which were identified by direct comparison of their retention times and UV spectra with those of both commercial standards (Sigma-Aldrich in compounds **1–5**, **9–12**) as well as internal (isolated and elucidated within CIBIS-IMSS, compounds **6**, **8**, and **9–12**). Subsequently, and specifically, the identity of the compounds called “kaempferol−3−O−glucopyranoside” (compound **7**) and “kaempferol−3−O−(3″,4″−Diacetyl−2″,6″−di−E−*p*−coumaroyl) −glucopyranoside” (compound **15**) were determined by both HPLC and NMR (see Supplementary Material Figures S1 and S2 and Figure 1). The identification of the terpenoids, β−amyrin (8 mg, **17**), β−sitosterol (12 mg, **18**), and ursolic acid (17 mg, **19**), was carried out by the analysis of the NMR data of ^1^H and ^13^C (see Supplementary Material Figures S3–S5).

**Figure 2:**
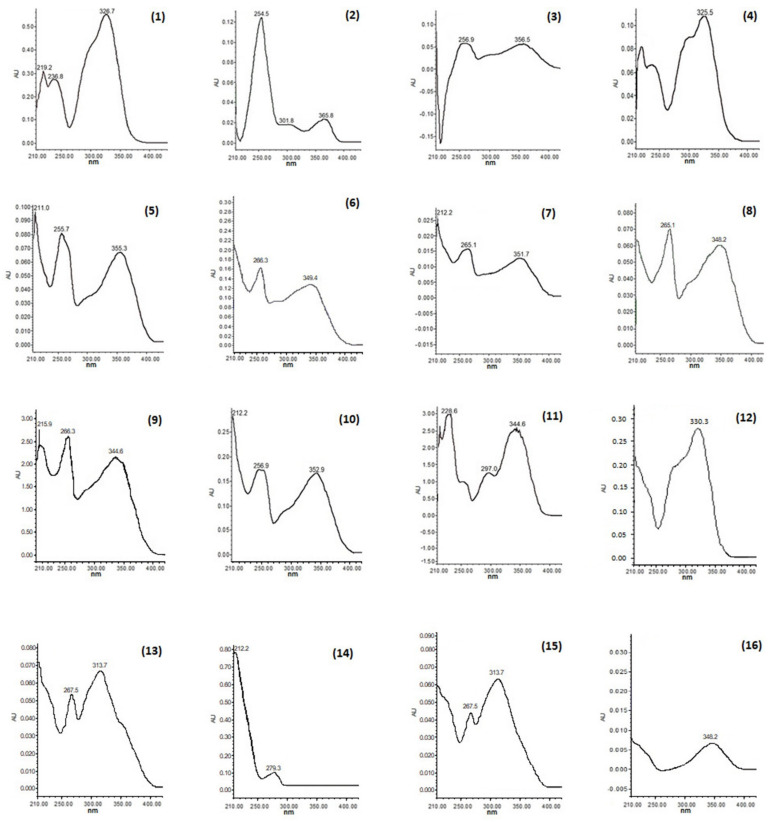
UV spectra of phenolic compounds (chlorogenic acid (**1**), coumaric acid (**2**), quercetin−3−O−rutinoside (**3**), caffeic acid (**4**), quercetin−3−O−glucoside (**5**), kaempferol−3−O−sophoroside (**6**), kaempferol−3−O−glucopyranoside (**7**), kaempferol−3−O−sambubioside (**8**), luteolin−7−O−glucoside (**9**), quercetin−3−O−rhamnoside (**10**), scopoletin (**11**), rosmarinic acid (**12**), tiliroside (**13**), coumaric acid derivative (**14**), kaempferol 3−O−(3″,4″−Diacetyl−2″,6″−di−E−*p*−coumaroyl)−glucopyranoside (**15**), and coumarin (**16**) in three studied species of white oak (*Q. rugosa*, *Q. obtusata,* and *Q. glabrescens*).

The richness of sm showed the following pattern: *Q. rugosa* (16) > *Q. glabrescens* (14) > *Q. obtusata* (11). The differences in the number of compounds found among oak species are due to the phenolic compounds, since the same number of terpenoid compounds was found in all three species (see Supplementary Material Figures S3–S5, Figure 2 and Table 1).

### 2.3. Identification of Species-Specific Compounds

Coumaric acid, tiliroside, and coumarates were only recorded in *Q. rugosa*, so these three sm may be considered species-specific; their presence has not been reported in other oak species, at least in Mexico. Likewise, rosmarinic acid and coumarins were only identified in *Q. glabrescens*, so this can also be considered a species-specific marker, making this the first time that its presence has been reported in oaks in Mexico. In contrast, luteolin−7−O−glucoside was only identified in *Q. obtusata*, so it can be considered a species-specific marker too. Again, this is the first time that its presence has been reported in oaks in Mexico. Regarding the compound called “kaempferol−3−O− (3″,4″−Diacetyl−2″,6″−di−E−*p*−coumaroyl)−glucopyranoside”, reference is made to a compound previously reported by [19,29] found in *Q. ilex*. This compound has only been reported in three species of oaks (all belonging to the *Quercus* section) worldwide and none in Mexico. Therefore, this group of compounds should be considered as a specific section since the three study species analyzed here belong to the same section, as previously mentioned.

### 2.4. Isolation and Structural Elucidation of Compounds Kaempferol−3−O−Glucopyranoside (**7**) and Kaempferol−3−(3″,4″−Diacetyl−2″,6″−di−E−p−Coumaroyl)−Glucopyranoside (**15**)

**Figure 3:**
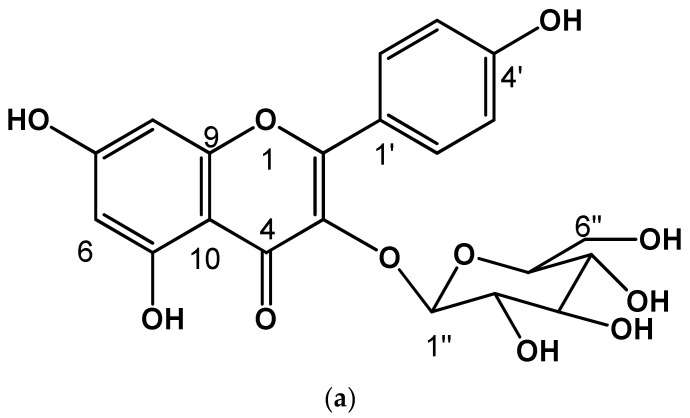

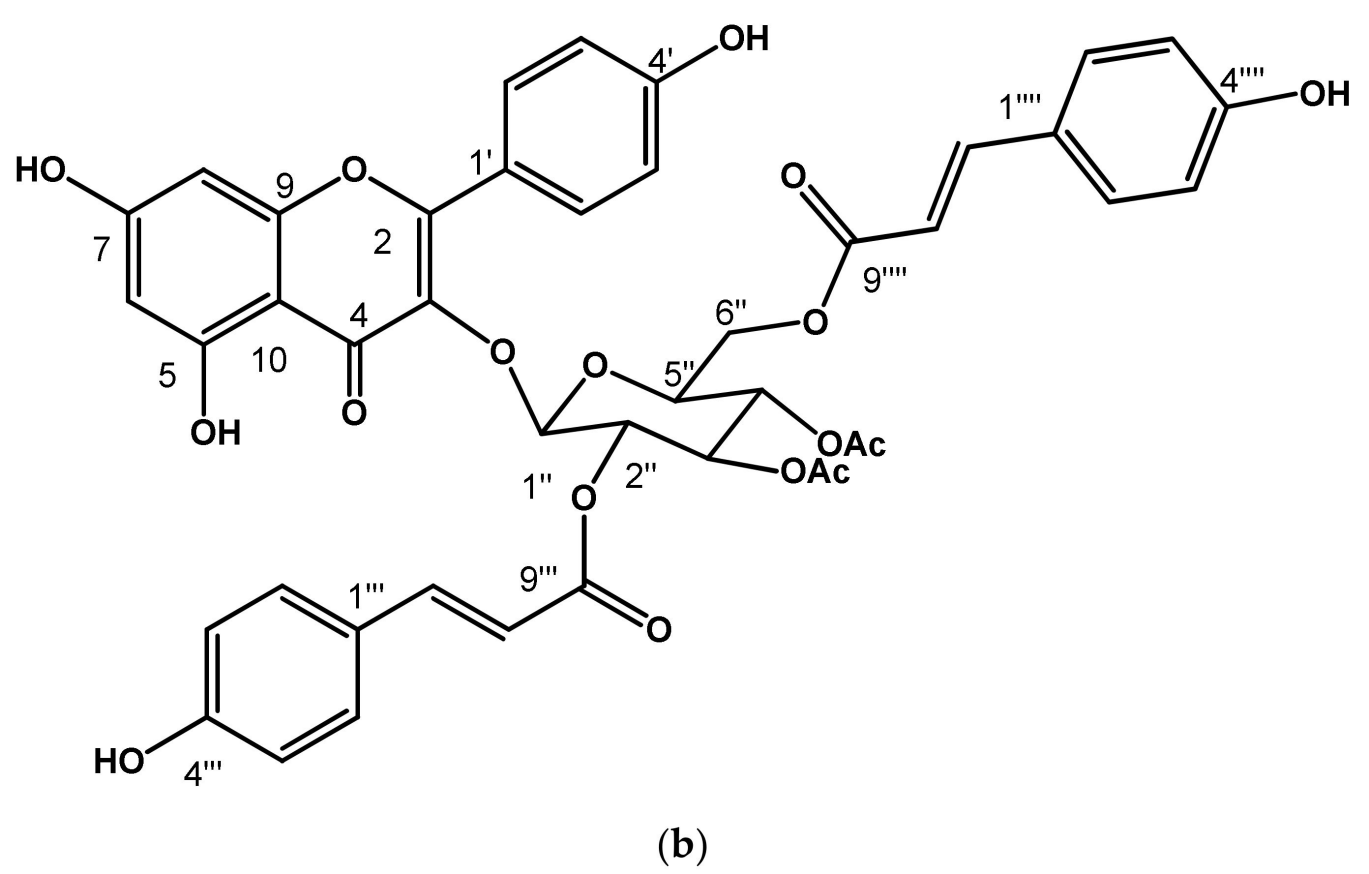
Chemical structure of identified flavonoids (**a**) Kaempferol−3−O−glucopyranoside, (**b**) Kaempferol−3−O−(3″,4″−Diacetyl−2″,6″−di−E−*p*−coumaroyl)−glucopyranoside (see Supplementary Material, Figures S1 and S2).

Compound (**7**) was isolated as a yellow powder. In the UV light spectrum, the compound showed a λmax 212, 265, and 351 nm, characteristic of a phenolic type of compound and identified as kaempferol−3−O−glucopyranoside. The comparison of the spectroscopic data with those described in the literature [30] allowed the determination of its chemical structure (see Figure 2 and Figure 3a).

Compound (**15**) was isolated as a yellow powder. In the UV light spectrum, the compound showed a λmax 201, 268 and 313 nm, characteristic of a phenolic type of compound (Figure 3b). In the ^1^H NMR spectrum, a doublet signal was observed at δ 6.03 (1H, d, J = 8.0 Hz, H-1″′) and with a chemical shift in ^13^C NMR at δ 99.6 (CH, C-1″) which corresponds to a ketal carbon of a sugar. This proton signal (δ 6.03, H-1″) correlated in the COSY experiment with the signal at δ 5.33 (1H, dd, J = 8.8, 9.1 Hz) was assigned to H-2′. Analysis of the homonuclear experiment (COSY) allowed us to identify the proton couplings of a hexose known as D-glucose. On the other hand, two broad signals characteristic of meta-coupled aromatic ring systems were observed in ^1^H NMR at δ 6.23 (1H, s, br) and 6.45 (1H, s, br) corresponding to H-6 and H- 8, respectively. Additionally, two other aromatic signals were observed at δ 8.07 (1H, d, 8.6 Hz, H-2′, H-6′) and 6.99 (1H, d, 8.8 Hz, H-3′, H-5′), corresponding to four aromatic protons, so it was determined that this skeleton corresponded to a flavonol known as kaempferol. This flavonol is bound in position one of glucose (δ 6.23 (1H, s, br) by the long-range correlation (HMBC) observed with the double bond signal at δ 133.8 assigned to C-3. And finally, the presence of two coumaroyl groups was observed by double bond hydrogen signals, as well as aromatic signals. According to the HMBC experiment, one of the coumaroyl groups is bound at the C-6″ position and the other at the C-2″ position) of the sugar. Thus, it was observed that the C-3″ and C-4″ positions of the sugar were found with acetate groups. Due to the previous analysis and the comparison of the data described [31], this molecule is called kaempferol 3−O−(3″,4″−Diacetyl−2″,6″−di−E−*p*−coumaroyl)−glucopyranoside (Figure 3b). This compound has already been isolated in the genus.

Kaempferol−3−O−glucopyranoside **(7)**: ^1^H NMR (CD_3_OD, 600 MHz); δ 6.19 (1H, d, 2.0 Hz, H-6), 6.38 (1H, d, H-8), 8.05 (1H, d, 8.3 Hz, H-2′, H-6′), 6.88 (1H, d, 8.3 Hz, H-3′, H-5′), 5.23 (1H, d, 8.3 Hz, H-1″), 3.42 (1H, dd, 7.6, 9.1 Hz, H-2″), 3.46 (1H, dd,8.3, 9.1 Hz, H-3″), 3.31 (1H, m, H-4″), 3.21 (1H, ddd, 2.2, 5.3, 7.6 Hz, H-5″), 3.69 (1H, dd, 2.2, 12.2 Hz, H-6a″), 3.55 (1H, dd, 5.3, 12.2 Hz, H-6b″); ^13^C NMR (CD_3_OD, 150 MHz); δ 159.0 (C, C-2), 135.4 (C, C-3), 179.4 (C, C-4), 163.0 (C, C-5), 100.1 (CH, C-6), 166.6 (C, C-7), 94.8 (CH, C-8), 158.5 (C, C-9), 105.5 (C, C-10), 122.8 (C, C-1′), 132.2 (CH, C-2′, C-6′), 116.0 (CH, C-3′, C-5′), 161.5 (C, C-4′), 104.1 (CH, C-1″), 75.7 (CH, C-2″), 78.0 (CH-C-3″), 71.3 (CH, C-4″), 78.4 (CH, C-5″), 62.6 (CH_2_, C-6″).

Kaempferol−3−O−(3″,4″−Diacetyl−2″,6″−di−E−*p*−coumaroyl)−glucopyranoside **(15)**: ^1^H NMR (CD_3_COCD_3_, 600 MHz); δ 6.23 (1H, s, br, H-6), 6.45 (1H, s, br, H-8), 8.07 (1H, d, 8.6 Hz, H-2′, H-6′), 6.99 (1H, d, 8.8 Hz, H-3′, H-5′), 6.03 (1H, d, 8.0 Hz, H-1″), 5.33 (1H, dd, 8.8, 9.1 Hz, H-2″), 5.50 (1H, dd, 9.1, 9.9 Hz, H-3″), 5.14 (1H, dd, 9.5, 9.9 Hz, H-4″), 4.09 (1H, m, H-5″), 4.18 (1H, d, br, 3.03 Hz, H-6a″), 4.16 (1H, d, 4.03 Hz, H-6b″), 7.56 (1H, d, 8.1 Hz, H-2″′, H-6″′), 6.90 (1H, d, 8.4 Hz, H-3″′, H-5″′), 7.72 (1H, d, 15.8 Hz, H-7″′), 6.36 (1H, d, 16.1 Hz, H-8″′), 7.46 (1H, d, 8.8 Hz, H-2″″, H-6″″), 6.90 (1H, d, 8.4 Hz, H-3″″, H-5″″), 7.48 (1H, d, 16.1 Hz, H-7″″), 6.19 (1H, d, 15.7 Hz, H-8″″), 2.02 (3H, s, COCH_3_-3″), 1.95 (3H, s, COCH_3_-4″); ^13^C NMR (CD_3_COCD_3_, 150 MHz); δ 157.8 (C, C-2), 133.8 (C, C-3), 178.4 (C, C-4), 160.8 (C, C-5), 99.6 (CH, C-6), 165.0 (C, C-7), 94.5 (CH, C-8), 158.2 (C, C-9), 105.5 (C, C-10), 122.4 (C, C-1′), 131.9 (CH, C-2′, C-6′), 116.0 (CH, C-3′, C-5′), 160.6 (C, C-4′), 99.6 (CH, C-1″), 72.6 (CH, C-2″), 73.5 (CH-C-3″), 72.8 (CH, C-4″), 69.6 (CH, C-5″), 62.4 (CH_2_, C-6″), 126.7 (C, C-1″′), 131.1 (CH, C-2″′, C-6″′), 116.6 (CH, C-3″′, C-5″′), 160.9 (C, C-4″′), 145.8 (CH, C-7″′), 114.7 (CH, C-8″′), 166.3 (C, C-9″′), 126.7 (C, C-1″″), 131.0 (CH, C-2″″, C-6″″), 116.7 (CH, C-3″″, C-5″″), 160.9 (C, C-4″″), 145.8 (CH, C-7″″), 114.7 (CH, C-8″″), 166.8 (C, C-9″″), 170.1 (C, 3″-COCH_3_), 170.2 (C, 4″-COCH_3_).

## 3. Discussion

Among the sm reported for species of *Quercus*, the most important in terms of diversity and representativeness are aliphatic, phenolic, and terpene compounds [19,20,21,22,23,24,25,26,27,29,32,33,34]. Consequently, in this study, we emphasized the determination of phenolic compounds since it has been documented that their production, as well as their heritability (a measure of the reproducibility of the phenotype within a set of genotypes [35]), are strongly determined by genetic factors [36,37,38,39,40,41,42], which reduce the influence of environmental factors.

In general, oak species present elevated values of genetic diversity, which is favored in part by recurring events of interspecific hybridization since oaks have weak reproductive barriers which favor gene flow among participating species [43]. In this sense, Castillo-Mendoza et al. [27] documented events of interspecific hybridization in *Q. rugosa*, *Q. glabrescens*, and *Q. obtusata*, which favor an increase in their levels of genetic diversity. For this reason, the richness of sm found in this study in each oak species (*Q. rugosa* 16 sm) > *Q. glabrescens* (14 sm) > *Q. obtusata* (11 sm) can be partly explained by the levels of genetic diversity gained from interspecific hybridization. The hybridization phenomenon can modify the genetic diversity of parental species and putative hybrid creating qualitative and quantitative variation in sm by altering biogenetic pathways. Various studies have documented that the production of sm is strongly regulated by the genetic information contained in the taxa where they are produced; see [21,26,27,40,44] for more details. Therefore, studies in the hybrid complex must consider that interspecific gene flow may promote synergistic or antagonistic relationships that positively or negatively impact the production of sm, promoting the occurrence of the following production patterns in hybrid plants: (1) a combination of parental species metabolites, (2) lack of parental metabolites, and (3) new metabolites which do not present in the parental species (44). The wide range of responses shown by the hybrids suggests that hybridization phenomenon may favor the formation of sm and/or that the mixture of metabolites may give new functions to existing ones.

For example, *Q. rugosa* is the species that has the widest geographic distribution, occupying both xeric and mesic environments (21 states in Mexico, [9]), which favor contact (sympatry) with different species of white oaks promoting interspecific hybridization events [27]. This could explain the fact that *Q. rugosa* is the species with the highest number of sm identified in Mexico: 36 in total, considering the sm described by Yarnes et al. [24] and resulting from this study. Gene flow via introgressive hybridization promotes the exchange of genetic material between *Q. rugosa* and participating species (*Q. depressipes*, *Q. laeta*, *Q. arizonica* [45], *Q. grabrescens* and *Q. obtusata* [27]), facilitating the incorporation of novel genetic material, which can increase the production of diverse sm.

On the other hand, *Q. glabrescens* is the species that presents the most restricted geographic distribution (eight states in Mexico, [9]), but the sympatry of *Q. glabrescens* with *Q. rugosa* and *Q. obtusata* has favored hybridization events in both species [27,28], which would explain why *Q. glabrescens* is the species with the second most sm in this study.

Finally, *Q. obtusata* is distributed in mesic and xeric environments and has an intermediate geographic distribution (18 states in Mexico, [9]) between *Q. rugosa* and *Q. glabrescens*. However, there is only one recorded hybridization between *Q. obtusata* and *Q. rugosa*, which could be a factor that limits the interspecific gene flow, reducing the ability to incorporate new genetic material, compared to the other oak species mentioned. This could explain the lower number of sm recorded for *Q. obtusata*.

In this study, we documented the presence of 19 compounds, 16 of which are grouped within the phenolic compounds and three of which belong to the terpenoids. The phenolic compounds **3**, **4**, **5**, and **10** were reported previously by Castillo-Mendoza et al. [27]. In general, our results are consistent with the most important sm groups reported for oaks worldwide, since of the 68 oak species studied in diverse parts of the world, 98.5% evaluated sm in leaves. Moreover, 8.9% (*n* = 6) of these species analyzed the sm in leaves and other tissue (i.e., corns, roots, and stem). In general, 1143 sm in oaks species between 1982 and 2021 have been documented (review for this article, unpublished data), and it was found that the most frequently reported sm are from the following groups, regardless of the tissue analyzed (leaves, corns, roots, and stem): flavonoids (49.34%), tannins (23.97%), terpenoids (13.56%), and others (13.13%, aliphatic compounds, carboxylic acids, fatty acids, organic acids, alkaloids, aldehydes, aliphatics, amino alcohols, sugars, benzofurans, heterocyclic compounds, organic compounds, steroids, glycerol, cardenolide glycosides, hydrocarbons, lipids, naphthofurans, saponins, and vitamins) [19,20,21,22,23,24,25,26,27,29,46,47,48].

For Mexican oaks, the phenolic compounds and terpenoids are again well represented. Of the 15 species studied, 48 distinct sm have been reported with: phenolics, glucoside flavonoids, proanthocyanidins, procyanodolics, tannins, and terpenoids [24,25,26,27]. In this study, 36.8% of the sm identified (scopoletin, ursolic acid, β−amyrin, luteolin−7−O−glucoside, kaempferol−3−O−sophoroside, kaempferol 3−O−glucopyranoside, and kaempferol−3−O−sambubioside) are new records for oaks worldwide, which demonstrates the need for more studies focused on characterizing the chemical profile of Mexican oak species. Even more so since Mexico is recognized as the main center of oak diversification worldwide [9].

Few studies have carried out a specific analysis of the metabolic profile of oak species, possibly because of the technical and economic difficulties as well as the lack of investigators pursuing this area of study. Furthermore, depending on the oak species studied, as well as the organs analyzed (branch, trunk, leaves, and fruits), the species show qualitative differences in the presence of their metabolic profiles [48,49,50,51,52,53], demonstrating the chemical complexity that oak species present in each of their structures, which significantly hinders the characterization of their specific chemical profile.

In this study, the compounds identified by analyzing the leaves have been related to curative and preventive medical functions (see Table 2). In general, the compounds identified in the three study species have different functions, which can be grouped as defenders of the organism that produces and/or consumes them. This can be explained by the fact that just over 84% are phenolic compounds and various studies have highlighted that one of the main characteristics of this group is their defensive function against various sources of stress, both biotic and abiotic [54,55,56,57,58]. Due to the multifunctional nature of the identified compounds, the four functions most frequently described for this type of sm were grouped together.

In particular, 84% of sm (**1–5**, **7**, **9–13**, **15–19**) have an antioxidant function. Various studies show that this type of compound normalizes antioxidant levels in blood and heart, increases/modulates the activity of enzymes, and reduces the presence of ROS and its uptake capacity, i.e., [61,63,64].

Similarly, 47% of the sm (**1–2**, **7**, **11–12**, **16–19**) have antibacterial functions, mainly against Gram-positive bacteria that have shown different degrees of resistance. In particular, the activity of these compounds has been compared with other antibacterial agents showing similar results, and their activity is related to the antioxidant activity of polyphenol oxidase to generate reactive quinone species, i.e., [60,83].

Furthermore, in 32% of the sm (**6**, **9**, **13**, **16**, **19**) anti-inflammatory activity has been reported. Said activity is modulated by the ability of these compounds to inhibit proinflammatory cytokines, inhibit the release of histamine, and reduce leukocyte levels, interleukin 1-beta, among other similar processes, i.e., [79,81].

In addition, 21% of these sm (**1–2**, **11**, **18**) show antifungal activity, which is related to the ability of these compounds to oxidize and thereby form quinones or show their power to inhibit germ tube development and/or the germination of the conidium, i.e., [65,77].

Finally, the similarity in the presence and production of the sm among the different oak species in this study may be related to the phylogenetic closeness of the species [12]. Thus, this may suggest that the species of white oaks under study have similar metabolic pathways. Another possible explanation is that given the recurrent introgressive hybridization events within *Quercus* [21,27,28,43], there may be constant interspecific gene flows that result in similar presence and production of many sm.

## 4. Materials and Methods

### 4.1. Equipment and Reagents

The methodology used in this study was based on that used by Ble-González et al. [83] but with some modifications. NMR spectra were recorded on an Agilent DD2-600 at 600 MHz for 1H and 150 MHz for ^13^C NMR, using CD_3_OD as the solvent. Chemical shifts are reported in ppm relative to TMS. Thin-layer chromatography (TLC) was performed using TLC Silica gel 60, F254, and 20 × 20 cm aluminum sheets (Merck KGaA, Darmstadt, Germany). High-performance liquid chromatography (HPLC, Waters, Milford, MA, USA) analyses were performed on a Waters 2695 Separation module system, equipped with a photodiode array detector (Waters Co. 2996) and Empower 3 software (Waters Corporation, Milford, MA, USA).

### 4.2. Plant Material

The aerial parts of *Q. rugosa*, *Q. glabrescens,* and *Q. obtusata* were obtained from 60 individuals (20 ind./site, 300 gr/site) in Coajomulco in the state of Morelos (19°2′3.5″ N, 99°11′54.1″ W), Tlaxco in the state of Tlaxcala (19°41′42.32″ N, 98°4′41.22″ W), and Chamilpa, in Morelos (18°59′32″ N, 99°13′50″ W), respectively. All the trees sampled were mature individuals that did not present any apparent damage. One sample of each species was prepared for taxonomic determination by Gabriel Flores Franco at the HUMO herbarium at the Autonomous University of the State of Morelos, with the following accession numbers, for *Q. rugosa*, *Q. obtusata*, and *Q. glabrescens* (Voucher No. 39790, 39791, and 39792, respectively). The fresh plant material collected for each oak species was dried in the shade at ambient temperature. All procedures were performed in accordance with permissions granted for plant sampling by the Mexican regulation SGPA/DGVS/004788/18.

### 4.3. Extracts

The dried and ground material (IKA-WERKE M20 mill) of each oak species (300 g/specie) was macerated in acetone (1 L/sample/triplicate) for 24 h at room temperature. It was then filtered (Whatman No. 4 Merck, Darmstadt, Germany) and concentrated in a rotoevaporator (BUCHI R-114, Flawil, Switzerland) at reduced pressure to obtain the acetonic extracts of *Q. rugosa* (eaQr), *Q. glabrescens* (eaQg), and *Q. obtusata* (eaQo), respectively. The chemical separation was carried out in a glass gravity column for each of the extracts. The acetonic extract (5 g) absorbed in silica gel was added to a glass column (30 × 1 cm) previously packed with 60 g of silica gel (mesh 70-230, Merck), using as the mobile phase n-hexane, and increasing the polarity with acetone. The separation was followed by thin layer chromatography (TLC). The presence of flavonoids and terpenes was confirmed using commercial standards (e.g., rutin, scopoletin, and usolic acid; Sigma-Aldrich, Bellefonte, PA, USA).

We used high performance liquid chromatography (HPLC), which consisted of a chromatographic system with a separation module (Waters 2696), a photodiode array detector (Waters 2996), and a Licrosphere^®^ column (100 rp-18, 250 × 4 mm, 5 µm). We used quercetin−3−O−rutinoside, quercetin−3−O−glucoside, quercetin−3−O−rhamnoside, caffeic acid, and kaempferol−3−O−glucoside, inter alia as reference standards. An HPLC method (flavonoids) was used to analyze the acetonic extracts, the fractions, and the compounds of the three oak species. The identification of each reported compound was achieved using a Supelcosil LC-F column (4.6 mm, 250 mm diameter, 5 μm particle size; Sigma-Aldrich). The mobile phase consisted of a mixture of 0.5% trifluoroacetic acid (Solvent A) and acetonitrile (solvent B). The gradient system employed was as follows: 0–1 min, 0% B; 2–4 min, 10% B; 5–7 min, 20% B; 8–14 min, 30% B; 15–18 min, 40% B; 19–22 min, 80% B; 23–26 min, 100% B; and 27–28 min, 0% B. The flow was maintained at 0.9 mL. The duration of the method was 30 min with a flow rate of 0.9 mL min-1, and the sample injection volume was 10 µL. We did a wavelength sweep from (λ) 200–600 nm. HPLC and NMR analysis was used for elucidating the identity of the pure compounds obtained (see Table 1).

### 4.4. Isolation and Identification of Compounds (**1–16**)

The chromatographic fractioning of the three acetone extracts (eaQr, eaQo, and eaQg) was carried out as follows (Column 1, normal phase): 5 g of extract was dissolved in acetone and adsorbed in 7 g of normal phase silica gel, later placed in a glass column (30 × 1.5 cm) packed with 60 g silica gel (70-230 mesh, Merck), and eluted with a gradient system of 100% dichloromethane and increasing the polarity by 5% with methanol. The collection volume of the fractions was 180 mL, which was concentrated in a rotatory evaporator. In eaQr, 43 fractions were obtained and were analyzed by TLC and pooled according to similarity in their compounds, in nine fractions. In addition, in eaQg, 38 fractions were obtained and analyzed by TLC and pooled according to similarity in their compounds, in nine fractions. Finally, in eaQo, 45 fractions were obtained and analyzed by TLC, and pooled according to similarity in their compounds, in 12 fractions.

Within the phenolic compounds identified by HPLC, the presence of two compounds (**7** and **15**) was detected for the first time. There was no prior commercial standard for Mexican oaks, so their isolation/purification and subsequent elucidation were proceeded by NMR.

In the case of *Q. rugosa*, with respect to the nine groupings, in group two 90:10, subfraction 8–13, the presence of compound **1** was determined, and in group four 80:20, subfraction 21–26, the presence of compound **2**. Subsequently, the precipitate from said column was analyzed, for which a second column was made in reverse phase (water-acetonitrile) taking the precipitate from group two. From said column, the subsequent fractions were analyzed in reversed phase. In group four 75:25, subfraction 21–26, the presence of compounds **3**, **4**, **5**, **10,** and **11** was determined. In group five, subfraction 27–33, the presence of compounds **6** and **8** was determined. In this group, a yellow powder was precipitated which, once purified, was analyzed by NMR, and identified as **7** (see Supplementary Material Figure S1, Figure 2 and Figure 3a). In group eight, subfraction 34–37, the presence of compounds **13** and **14** was determined. In addition, within this group and once the two previously mentioned compounds were identified, a yellow precipitate was obtained that was analyzed in a following reverse phase column, which resulted in the identification by NMR of a compound that was identified as **15** (see data Supplementary Material Figure S2, Figure 2 and Figure 3b).

For *Q. glabrescens*, with respect to the nine groupings, in group three 90:10, subfraction 9–12, the presence of **1** was determined. Subsequently, the precipitate from said column was analyzed, for which a second column was carried out in reverse phase (water-acetonitrile) taking the precipitate from group three. From said column, the subsequent fractions were analyzed in reversed phase. In group six, subfraction 23–27, the presence of compounds **3**, **4**, **5**, **10,** and **11** was determined. In group seven, subfraction 28–30, the presence of compounds **6**, **8**, and **12** was determined. In this group, a yellow powder was precipitated which, once purified, was analyzed by NMR and identified as **7**. In group eight, subfraction 75, a coumarin (**16**) was determined. In addition, within this group and once this compound was identified, a yellow precipitate was obtained that was analyzed in a following reverse phase column which resulted in the identification by RNM of a compound that was identified as **15**.

Finally, for *Q. obtusata*, within the 12 groupings, compounds were only identified in the reverse phase columns (water-acetonitrile) of group five, subfraction 21–26, where the presence of compounds **3**, **5**, **10,** and **11** was determined. In meeting eight, subfraction 27–30, the presence of compound **8** was determined. In said group, a yellow powder was precipitated which, once purified, was analyzed by NMR, and identified as **7**. In meeting nine, subfraction 68, a yellow precipitate was obtained that was analyzed in a subsequent reverse phase column which resulted in the identification by NMR of a compound that was identified as **15**. It should be noted that, except for compounds **7** and **15**, all the others were only identified by HPLC in the three study species.

### 4.5. Isolation and Identification of Compounds (**17–19**)

The presence of the terpenes known as β-sitosterol (**17**), β-amyrin (**18**), and ursolic acid (**19**) was established by TLC in the crude extracts obtained directly from the maceration by direct comparison with the standards. Subsequently, the three compounds were isolated by normal phase column chromatography, and finally, the specific identity of each was confirmed by NMR (see Supplementary Material Figures S3–S5). These compounds were found to be present in all three oak species.

## 5. Conclusions

The characterization of the nineteen specialized metabolites found in *Q. rugosa*, *Q. glabrescens*, and *Q. obtusata*, of which 36.8% (*n* = 7) represent new records for oak species worldwide, revealed the importance of increasing the study of oaks. In particular, Mexico is considered one of the centers of diversification of the genus *Quercus*. Therefore, it is relevant to generate knowledge of the chemical profile and their anthropocentric and phytocentric roles, in addition to the importance that these compounds may have from the ecological, physiological, pharmacological, and commercial points of view. Remarkably, the compounds identified in this study have been related to various pathologies of medical importance. It is essential to continue working on the chemical characterization of more species of oaks that provide alternatives to a multi-resistance scenario and the appearance of new diseases that impact human and ecosystem health.

## Figures and Tables

**Table 1:** Comparison of phenolic compounds (**1–16**) identified in *Q. rugosa*, *Q. obtusata* and *Q. glabrescens*.

	Study Species				
	*Q. rugosa*	*Q. obtusata*	*Q. glabrescens*	Retention Time	Identification Technique
Compound	[% au]	[% au]	[% au]	(min)	
**1** chlorogenic acid	(3.06)	ND	(1.38)	8.601	HPLC
**2** coumaric acid	(2.13)	ND	ND	8.834	HPLC
**3** quercetin−3−O−rutinoside	(2.64)	(2.39)	(1.11)	9.071	HPLC
**4** caffeic acid	(2.20)	ND	(0.56)	9.226	HPLC
**5** quercetin−3−O−glucoside	(0.43)	(0.05)	(1.14)	9.530	HPLC
**6** kaempferol−3−O−sophoroside	(1.85)	ND	(8.20)	9.681	HPLC
**7** kaempferol−3−O−glucopyranoside	(0.12)	(0.38)	(0.14)	9.715	NMR
**8** kaempferol−3−O−sambubioside	(25.83)	(87.28)	ND	9.816	HPLC
**9** luteolin−7−O−glucoside	ND	(0.40)	ND	9.340	HPLC
**10** quercetin−3−O−rhamnoside	(5.03)	(7.63)	(5.11)	10.067	HPLC
**11** scopoletin	(0.11)	(0.09)	(0.14)	10.524	HPLC
**12** rosmarinic acid	ND	ND	(3.32)	10.973	HPLC
**13** tiliroside	(1.48)	ND	ND	12.516	HPLC
**14** coumaric acid derivative	(3.75)	ND	ND	25.315	HPLC, only UV spectrum
**15** kaempferol−3−O− (3″,4″−Diacetyl−2″,6″−di−E−*p*−coumaroyl)−glucopyranoside	(51.31)	(1.76)	(74.18)	27.932	NMR
**16** coumarin	ND	ND	(4.70)	29.016	HPLC, only UV spectrum

(ND): not detected; bolt number = species-specific compounds.

**Table 2:** Medical functions reported for each majority compound purified from three white oak species (*Q. rugosa*, *Q. obtusata,* and *Q. glabrescens*) from the Trans-Mexican Volcanic Belt.

Specialized Metabolites	Function	Reference
ursolic acid	Antioxidants, antibacterial, anti-inflammatory, antiparasitic, antiviral, anticancer	[59,60,61]
β−amyrin	Antioxidant, antibacterial, antifungal	[62]
β−sitosterol	Antioxidant, antibacterial, antiparasitic	[63]
chlorogenic acid	Antioxidant, antibacterial, antifungal, antiviral	[64]
coumaric acid	Antioxidant, antibacterial, antifungal	[65,66]
quercetin−3−O−rutinoside	Antioxidant	[67]
caffeic acid	Antioxidant	[68]
quercetin−3−O−glucoside	Antioxidant	[69]
kaempferol−3−O−sophoroside	Anti-inflammatory	[70]
kaempferol−3−O−glucopyranoside	Antioxidant, antibacterial, anti-inflammatory, anti-cancer, cardioprotective, neuroprotective, antidiabetic, anti-osteoporotic, estrogenic/antiestrogenic, anxiolytic, analgesic, antiallergic	[71]
kaempferol−3−O−sambubioside	Gastroprotective	[72]
luteolin−7−O−glucoside	Antioxidant, anti-inflammatory	[73]
quercetin−3−O−rhamnoside	Antioxidant, antiviral	[74,75]
scopoletin	Antioxidant, antibacterial, antifungal	[76]
rosmarinic acid	Antioxidant, antibacterial, antiviral, anti-carcer, anti-allergic, anti-thrombotic	[77]
tiliroside	Antioxidant, anti-inflammatory, anti-diabetic, cytoprotective, anti-cancer, antineoplastic, anti-hemorrhagic and antithrombotic activities	[78,79,80,81]
coumarate	Currently no reported medical functions	
kaempferol acetyl glucoside	Antioxidant	[82]
coumarin	Antioxidant, antibacterial, anti-inflammatory, anti-cancer, anti-coagulant, anti-platelet	[83]

## Data Availability

Data is contained within the article and Supplementary Materials.

## Supplementary Materials

The following supporting information can be downloaded at: https://www.mdpi.com/article/10.3390/plants11192610/s1, Figure S1a: Chemical structure, HPLC chromatogram and UV light spectrum of kaempferol−3−O−glucopyranoside; Figure S1b: ^1^H-NMR (CD_3_OD, 600 MHz) of kaempferol−3−O−glucopyranoside; Figure S1c: ^13^C-NMR (CD_3_OD, 150 MHz) of kaempferol−3−O−glucopyranoside; Figure S1d: ^1^H-^1^H(COSY)-NMR (CD_3_OD, 600 MHz) of kaempferol−3−O−glucopyranoside; Figure S1e: ^1^H-^13^C (HSQC) NMR (CD_3_OD, 600 MHz) of kaempferol−3−O−glucopyranoside; Figure S1f: ^1^H-^13^C (HMBC) NMR (CD_3_OD, 600 MHz) of kaempferol−3−O−glucopyranoside. Figure S2a: Chemical structure, HPLC chromatogram and UV light spectrum of kaempferol 3−O− (3″,4″−Diacetyl−2″,6″−di−E−*p*−coumaroyl)−glucopyranoside; Figure S2b: ^1^H-NMR (CD_3_OD, 600 MHz) of kaempferol−3−O−(3″,4″−Diacetyl−2″,6″−di−E−*p*−coumaroyl)−glucopyranoside; Figure S2c: ^13^C-DEPTQ-NMR (CD_3_OD, 150 MHz) of kaempferol−3−O−(3″,4″−Diacetyl−2″,6″−di−E−*p*−coumaroyl)−glucopyranoside; Figure S2d: ^1^H-^1^H (COSY) NMR (CD_3_OD, 600 MHz) of kaempferol−3−O−(3″,4″−Diacetyl−2″,6″−di−E−*p*−coumaroyl)−glucopyranoside; Figure S2e: ^1^H-^13^C (HSQC) NMR (CD_3_OD, 600 MHz) of kaempferol−3−O−(3″,4″−Diacetyl−2″,6″−di−E−*p*−coumaroyl)−glucopyranoside; Figure S2f: ^1^H-^13^C (HMBC) NMR (CD_3_OD, 600 MHz) of kaempferol−3−O−(3″,4″−Diacetyl−2″,6″−di−E−*p*−coumaroyl)−glucopyranoside. Figure S3a: TLC evidencing the presence of β−sitosterol in the three study species, ^1^H-NMR spectrum (600 MHz, CDCl_3_) of β-sitosterol; Figure S3b: ^13^C-NMR spectrum (150 MHz, CDCl_3_) of β-sitosterol; Figure S3c: ^1^H-^1^H NMR spectrum (COSY, 600 MHz, CDCl_3_) of β-sitosterol; Figure S3d: ^1^H-^13^C NMR spectrum (HSQC, 600 MHz, CDCl_3_) of β-sitosterol. Figure S4: TLC evidencing the presence of β−amyrin in the three study species, ^1^H-NMR Spectrum (600 MHz, CD_3_COCD_3_) of β−amyrin. Figure S5: TLC evidencing the presence of usolic acid in the three study species, ^1^H-NMR spectrum (600 MHz, CDCl_3_) of usolic acid.
