# Supplementary material for: Chemical Constituents and Their Production in Mexican Oaks (Q. Rugosa, Q. Glabrescens and Q. Obtusata)
Source: Plants (Basel). 2022 Oct 4;11(19):2610. doi: 10.3390/plants11192610 (PMC9573139; doi:10.3390/plants11192610)
Supplement: Supplementary file 1 [file plants-11-02610-s001.zip › plants-1941449-supplementary.pdf]

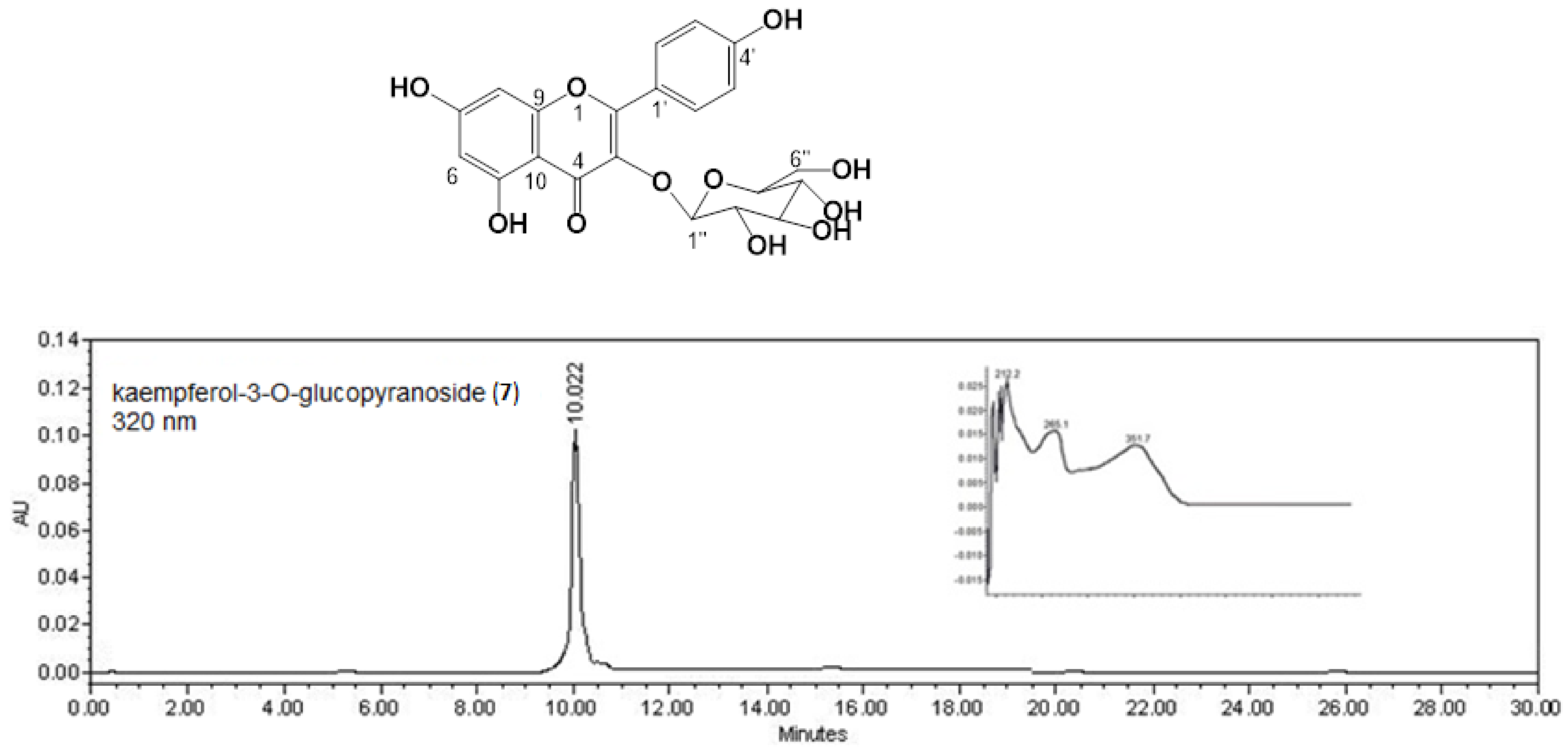

OH
4'
HO
9
O
1
1'
6
10
4
O
6''
OH
OH
OH
OH
O
1''
OH
O
kaempferol-3-O-glucopyranoside (7)
320 nm
10.022
AU
0.14
0.12
0.10
0.08
0.06
0.04
0.02
0.00
0.00
2.00
4.00
6.00
8.00
10.00
12.00
14.00
16.00
18.00
20.00
22.00
24.00
26.00
28.00
30.00
Minutes

Figure S1a

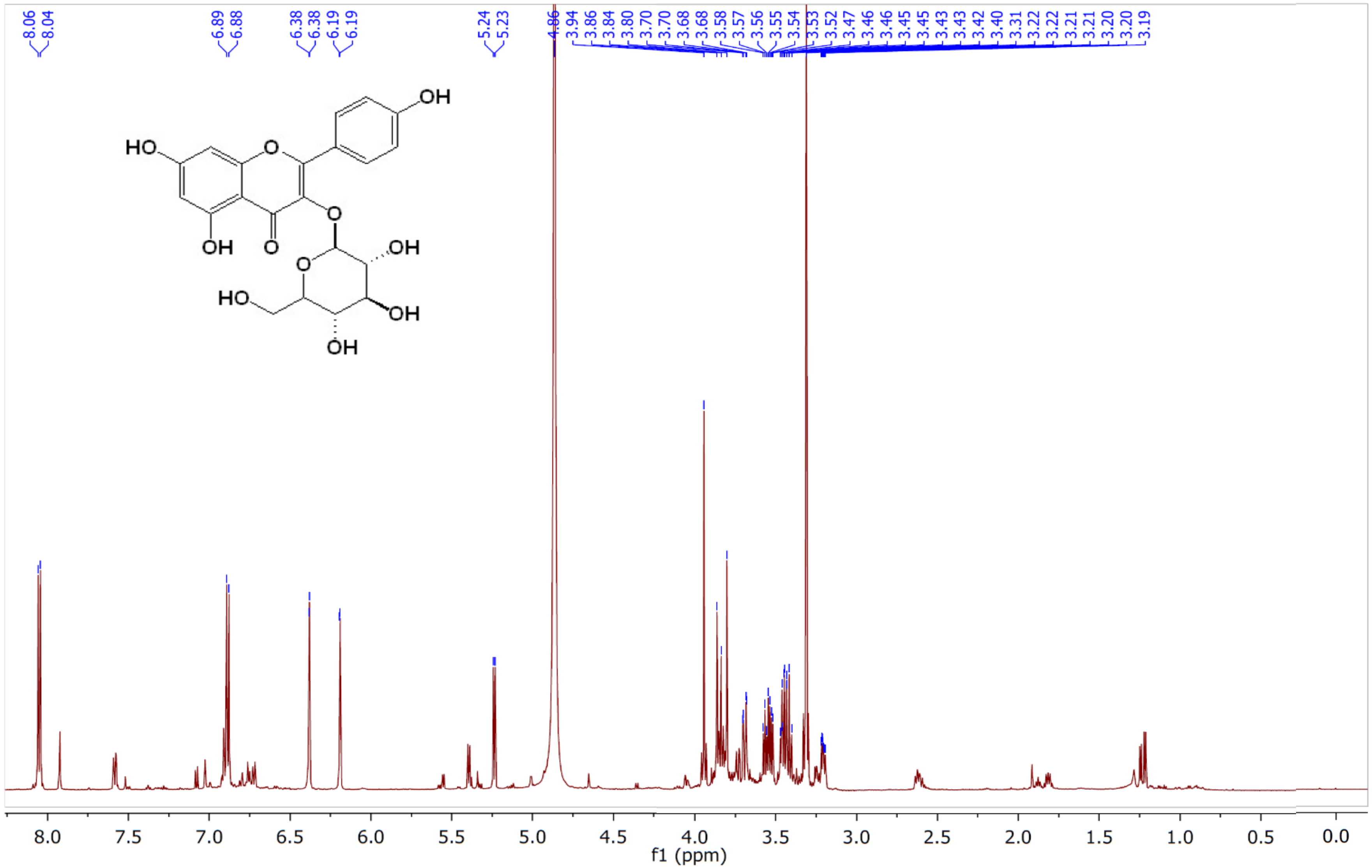
8.06
8.04
6.89
6.88
6.38
6.38
6.19
6.19
5.24
5.23
4.86
3.94
3.86
3.84
3.80
3.70
3.70
3.68
3.68
3.58
3.57
3.56
3.55
3.54
3.53
3.52
3.47
3.46
3.45
3.45
3.43
3.43
3.42
3.40
3.31
3.22
3.22
3.21
3.21
3.20
3.20
3.19
HO
OH
OH
HO
OH
OH
OH
f1 (ppm)

Figure S1b

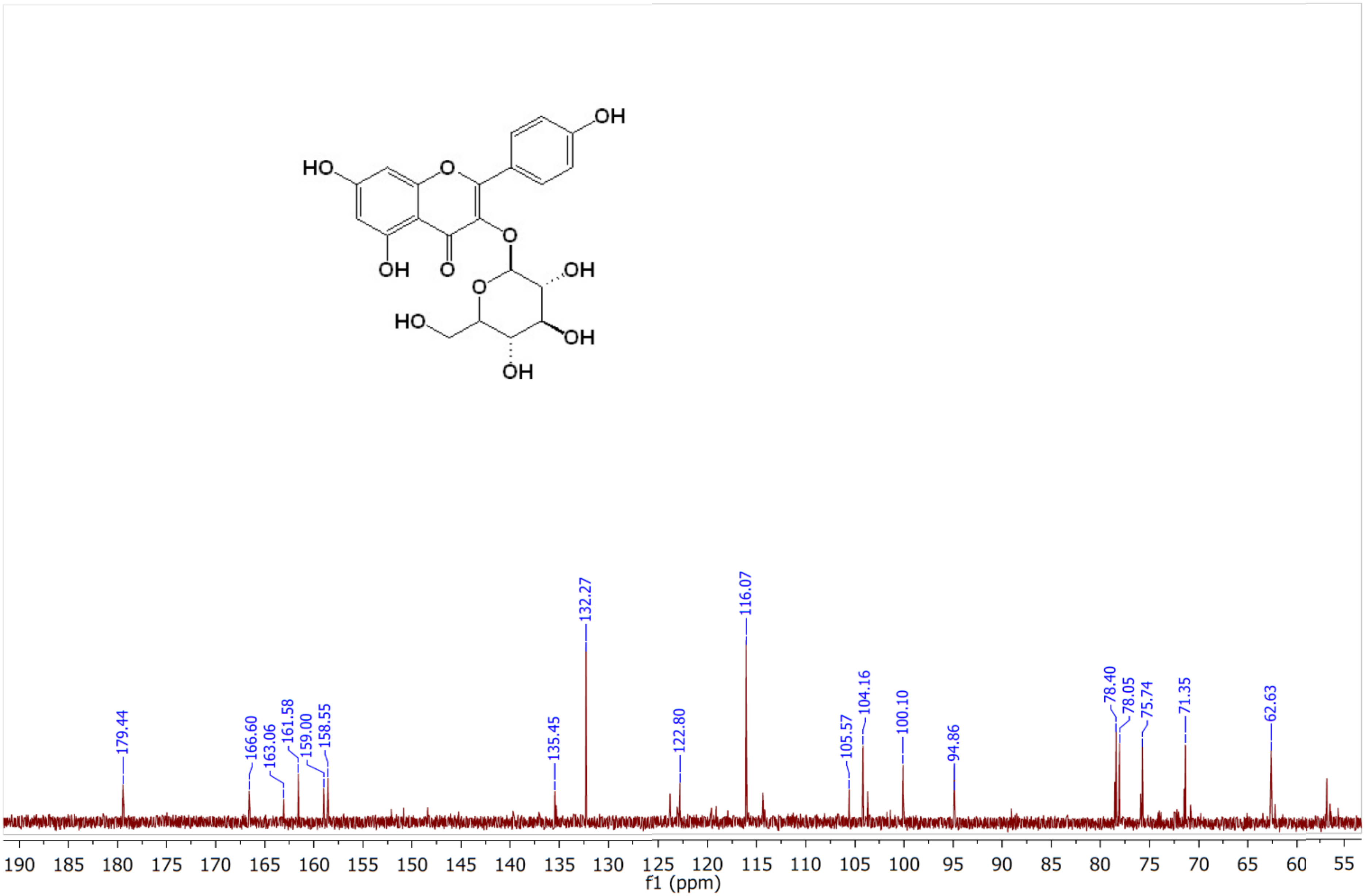
HO
OH
O
OH
O
O
OH
O
HO
OH
OH
179.44
166.60
163.06
161.58
159.00
158.55
135.45
132.27
122.80
116.07
105.57
104.16
100.10
94.86
78.40
78.05
75.74
71.35
62.63
190 185 180 175 170 165 160 155 150 145 140 135 130 125 120 115 110 105 100 95 90 85 80 75 70 65 60 55
f1 (ppm)

Figure S1c

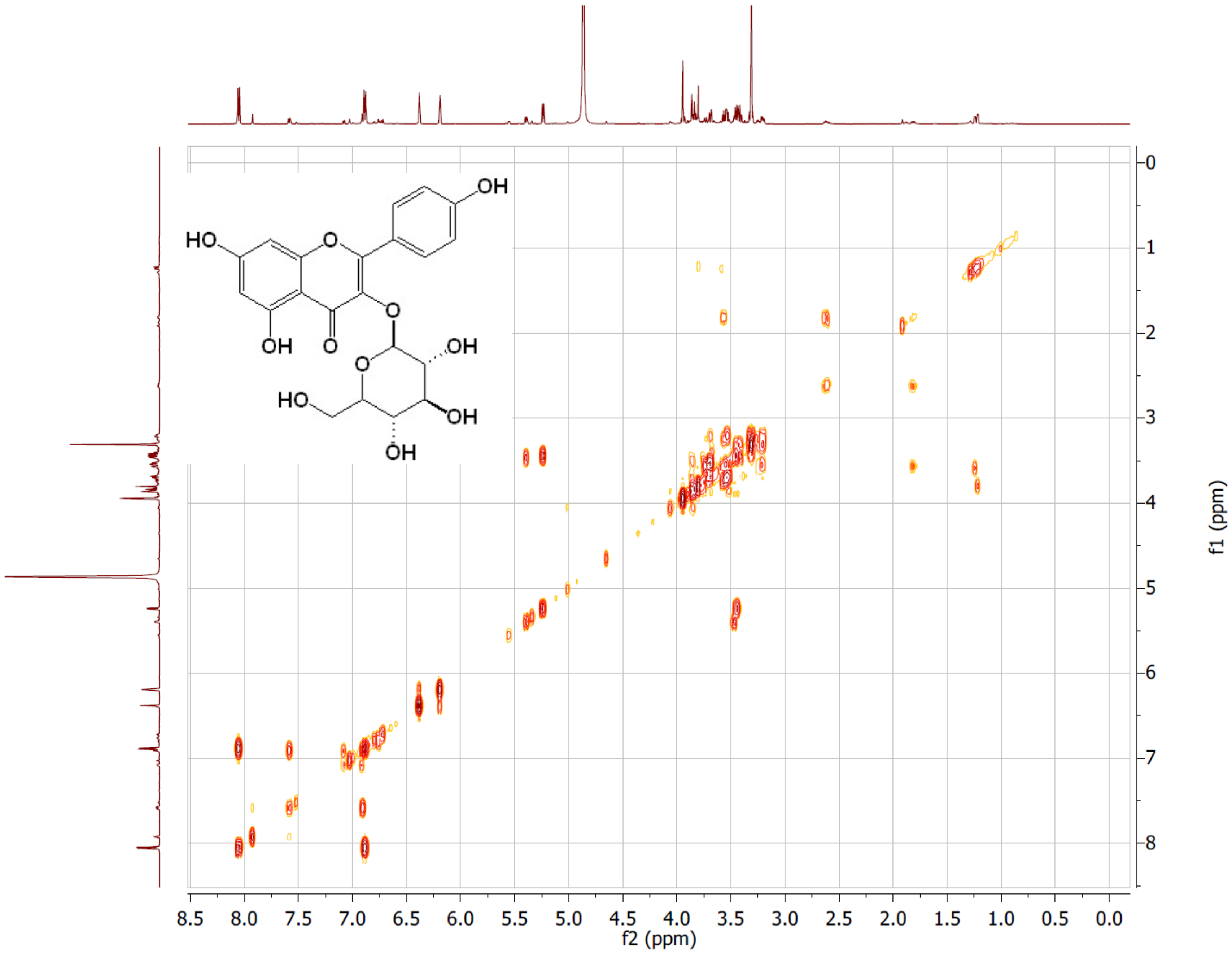

Figure S1d

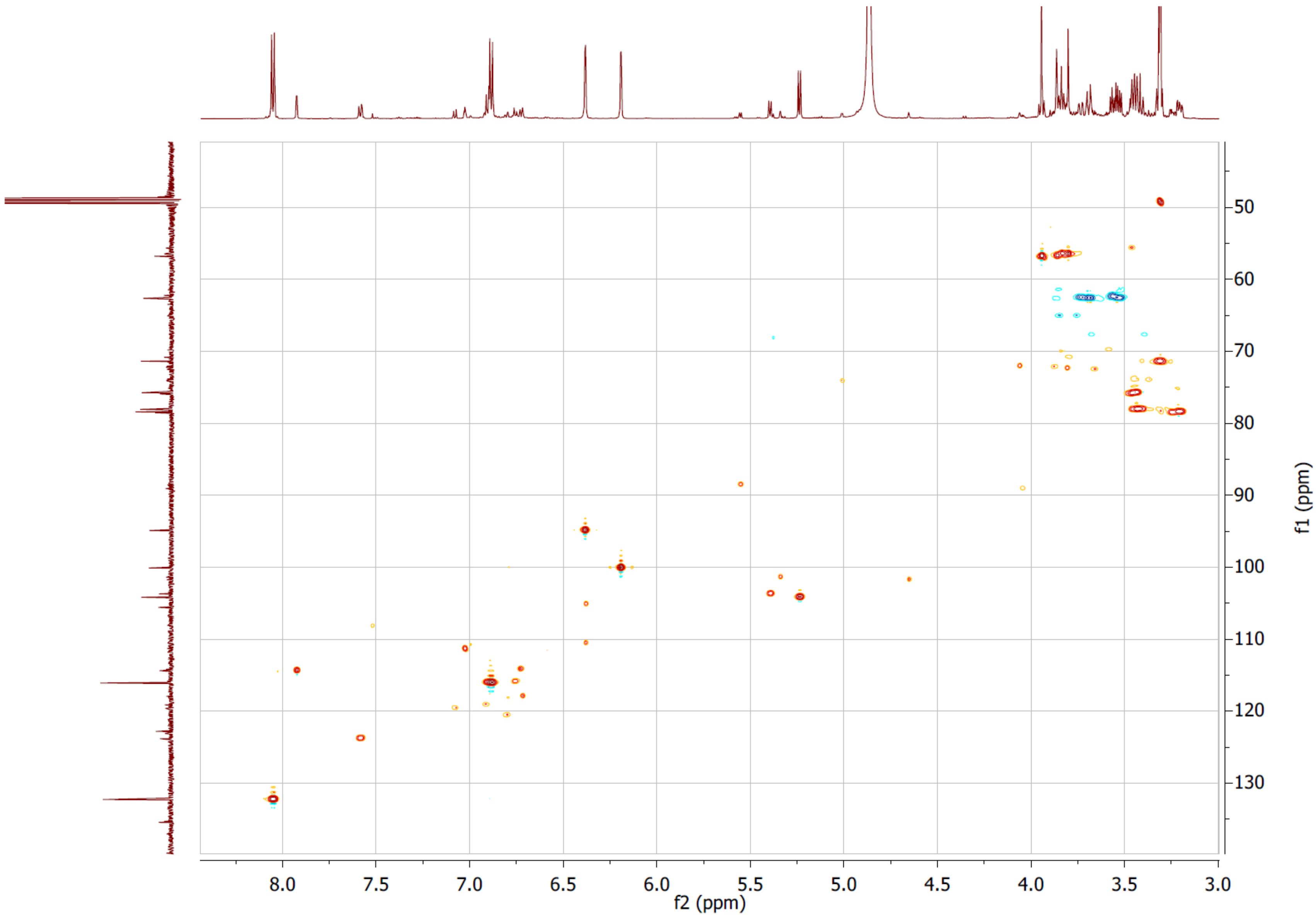

8.0
7.5
7.0
6.5
6.0
5.5
5.0
4.5
4.0
3.5
3.0
f2 (ppm)
50
60
70
80
90
100
110
120
130
f1 (ppm)

Figure S1e

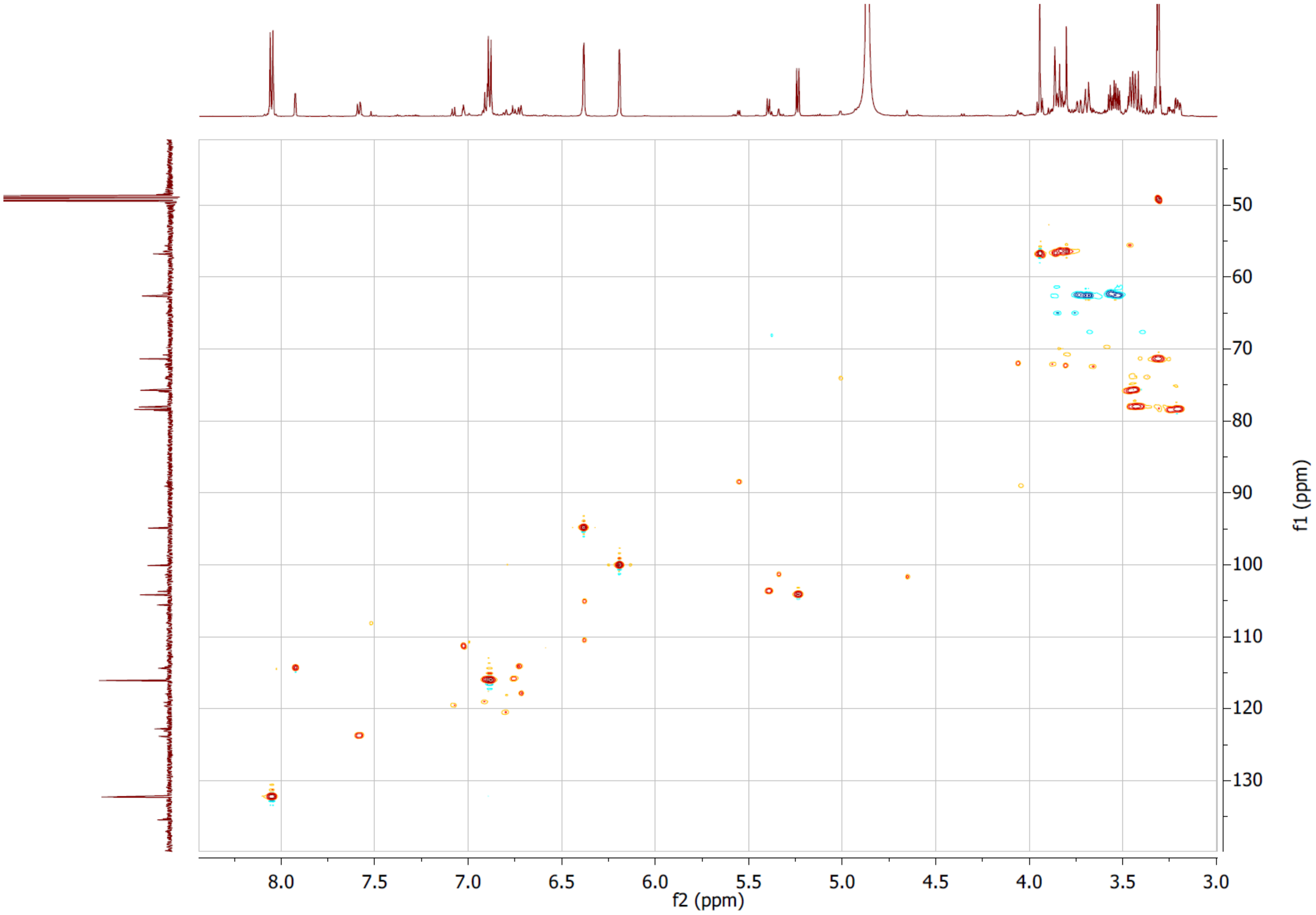
f1 (ppm)
f2 (ppm)
50
60
70
80
90
100
110
120
130
8.0
7.5
7.0
6.5
6.0
5.5
5.0
4.5
4.0
3.5
3.0

Figure S1f

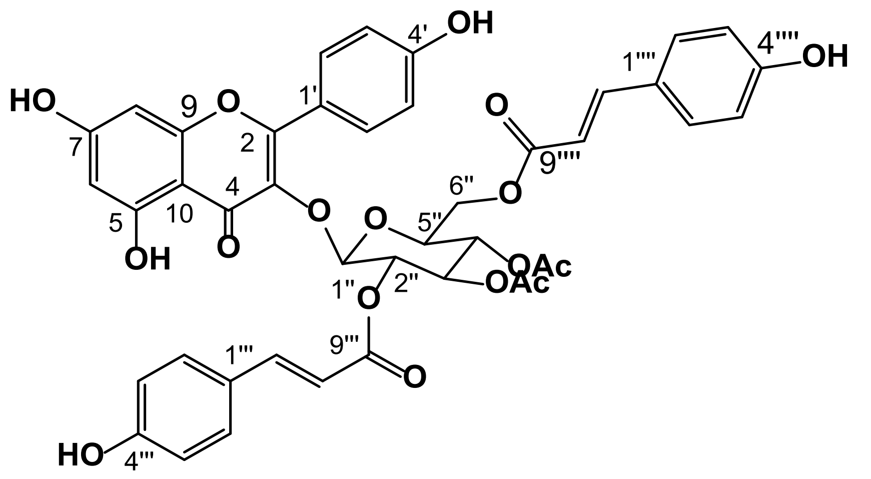

HO
OH
OH
OH
O
O
O
O
O
OAc
OAc
HO
4'
1'
9
2
7
4
10
5
6''
5''
1''
2''
1''''
4''''
9''''
1'''
9'''
4'''

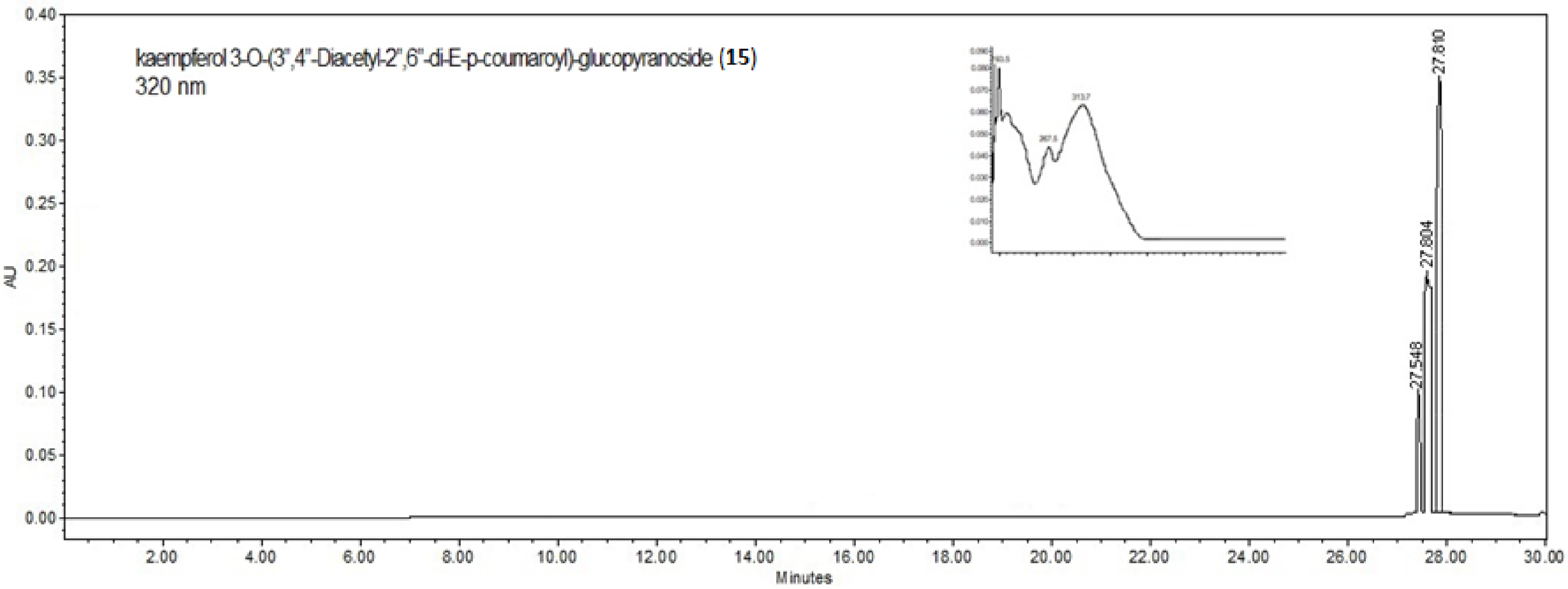

kaempferol 3-O-(3'',4''-Diacetyl-2'',6''-di-E-p-coumaroyl)-glucopyranoside (15)
320 nm
AU
Minutes
0.40
0.35
0.30
0.25
0.20
0.15
0.10
0.05
0.00
2.00
4.00
6.00
8.00
10.00
12.00
14.00
16.00
18.00
20.00
22.00
24.00
26.00
28.00
30.00
27.548
27.804
27.810

Figure S2a

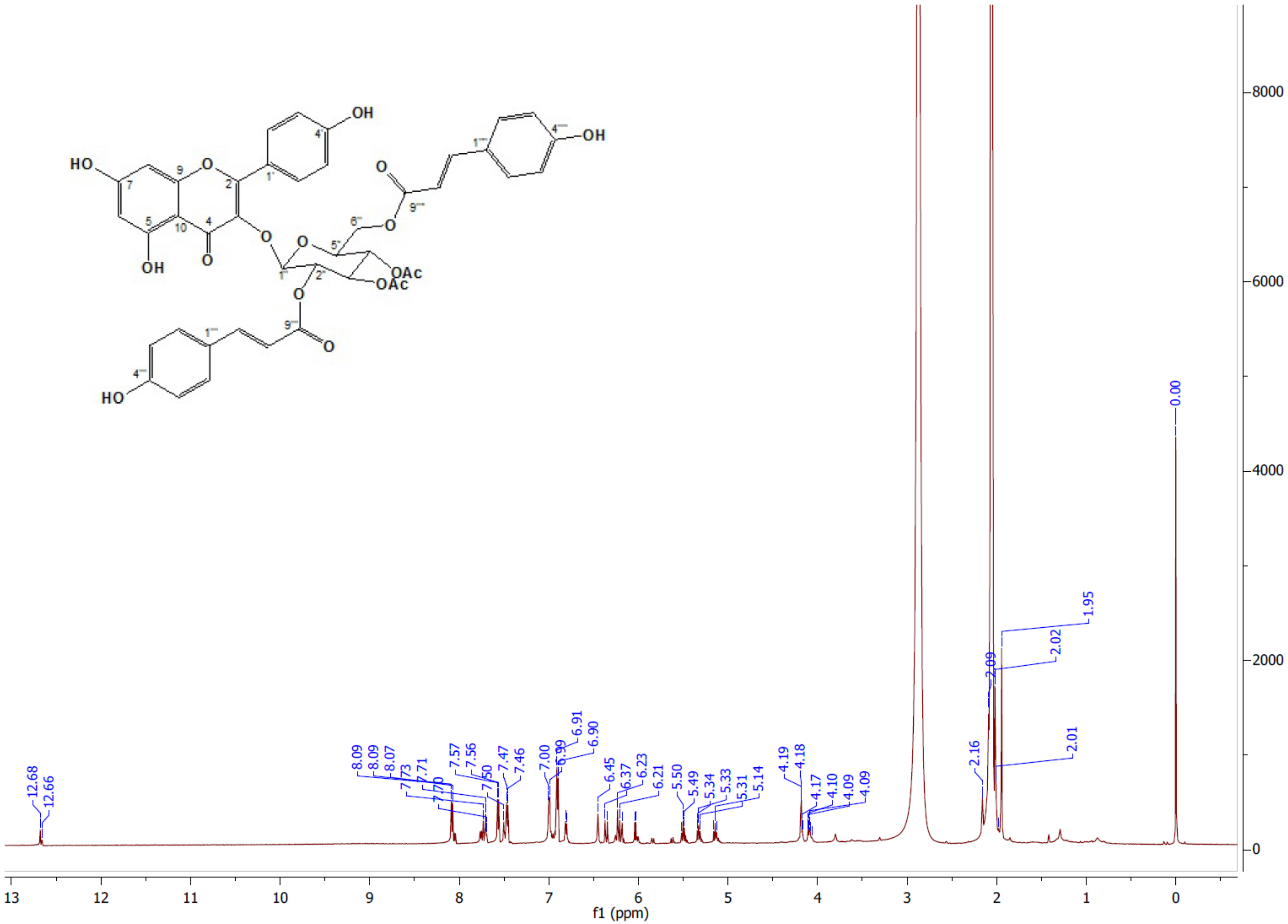
HO
OH
OAc
OAc
f1 (ppm)
13
12
11
10
9
8
7
6
5
4
3
2
1
0
8000
6000
4000
2000
0
12.68
12.66
8.09
8.09
8.07
7.73
7.71
7.70
7.57
7.56
7.50
7.47
7.46
7.00
6.99
6.91
6.90
6.45
6.37
6.23
6.21
5.50
5.49
5.34
5.33
5.31
5.14
4.19
4.18
4.17
4.10
4.09
4.09
2.16
2.09
2.02
2.01
1.95
0.00

Figure S2b

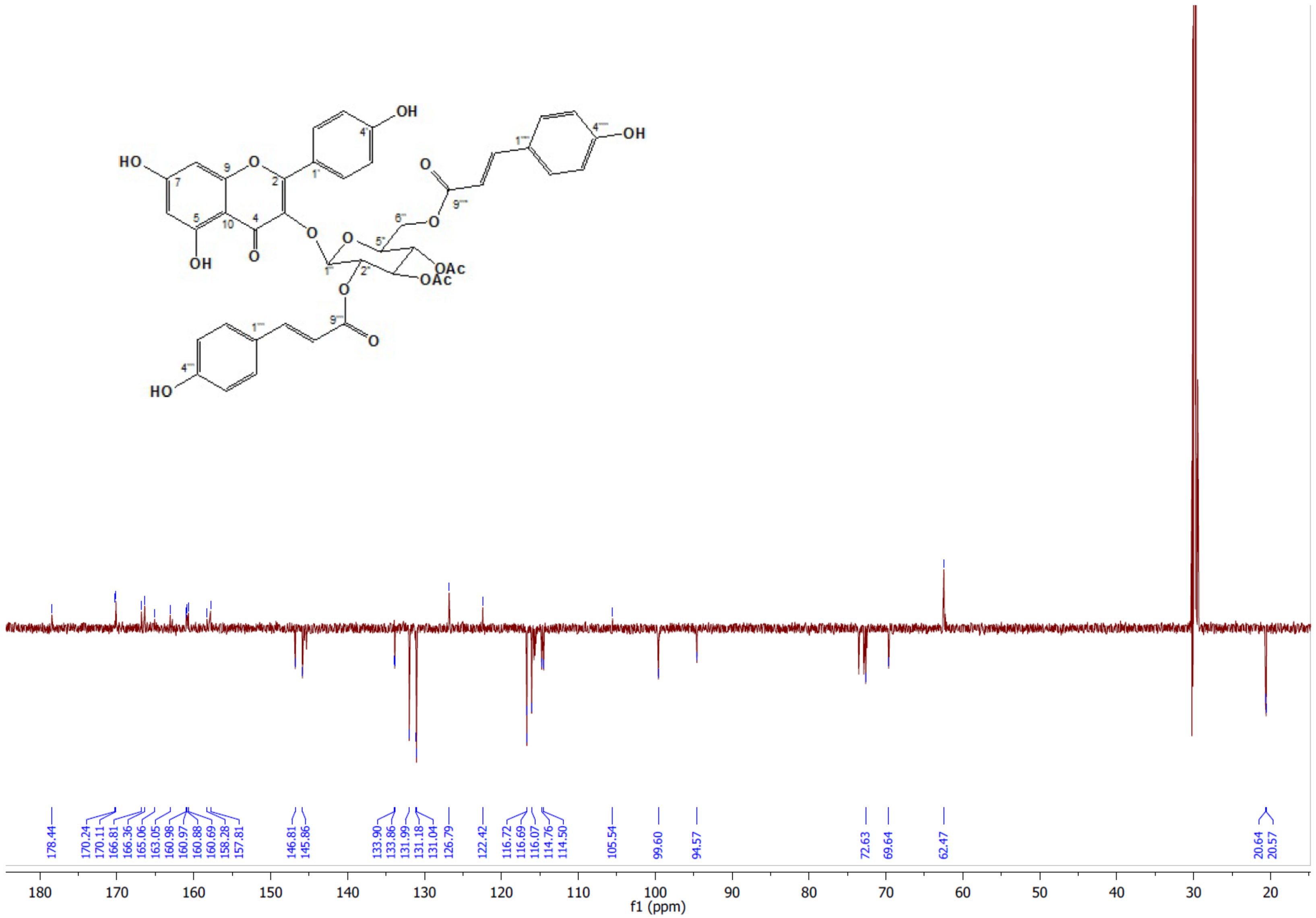

Figure S2c

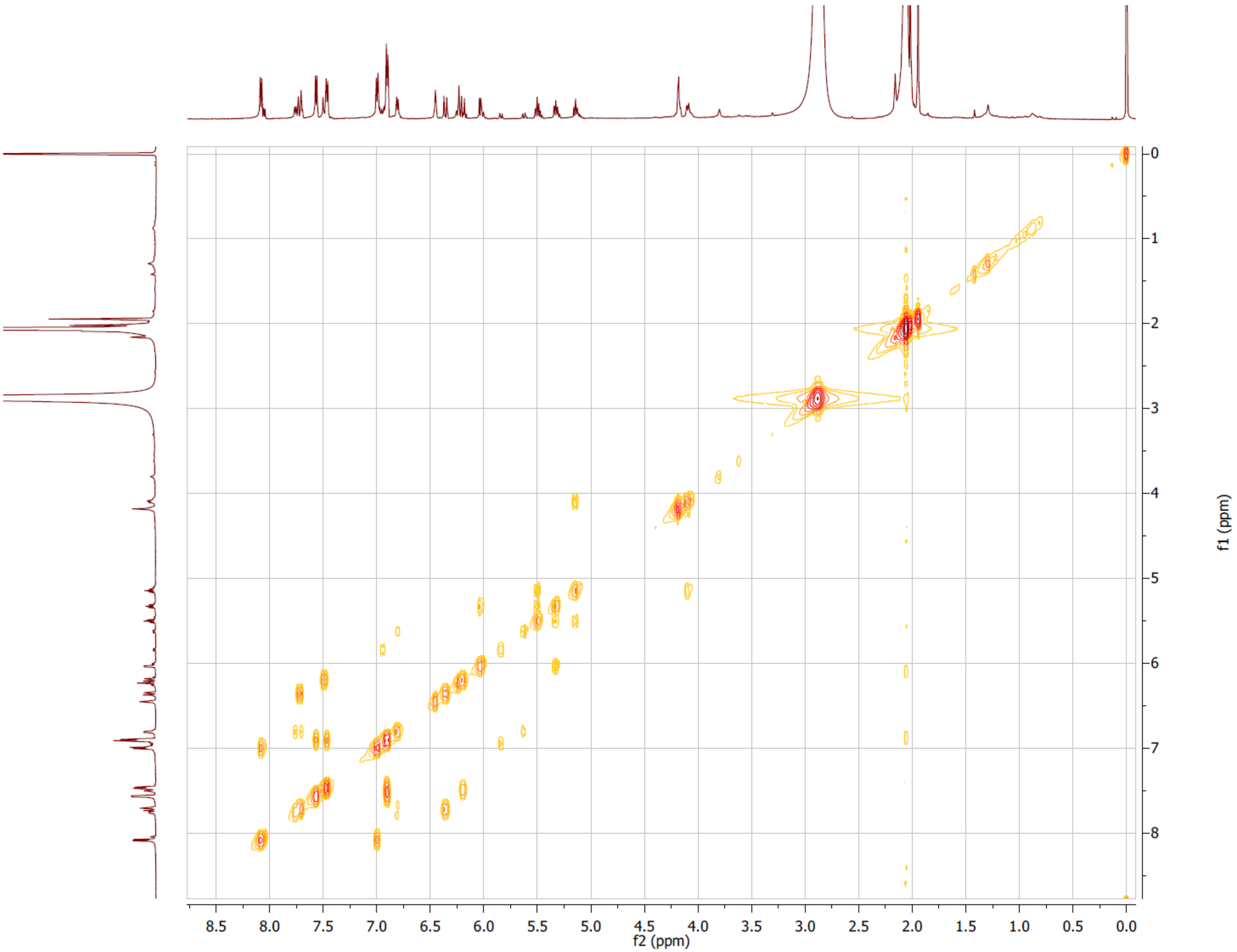

f1 (ppm)
f2 (ppm)
8.5
8.0
7.5
7.0
6.5
6.0
5.5
5.0
4.5
4.0
3.5
3.0
2.5
2.0
1.5
1.0
0.5
0.0
0
1
2
3
4
5
6
7
8

Figure S2d

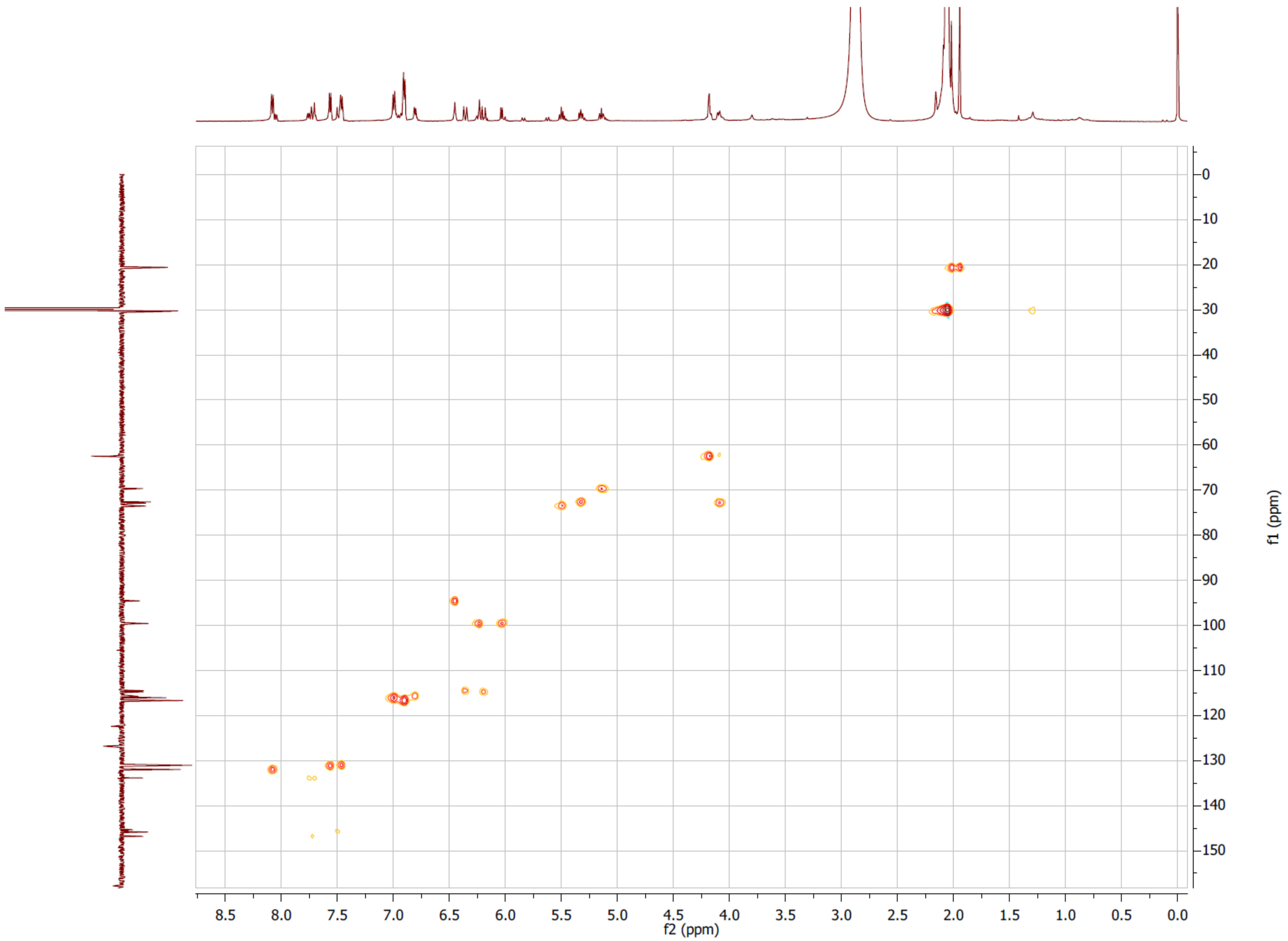

8.5
8.0
7.5
7.0
6.5
6.0
5.5
5.0
4.5
4.0
3.5
3.0
2.5
2.0
1.5
1.0
0.5
0.0
f2 (ppm)
0
10
20
30
40
50
60
70
80
90
100
110
120
130
140
150
f1 (ppm)

Figure S2e

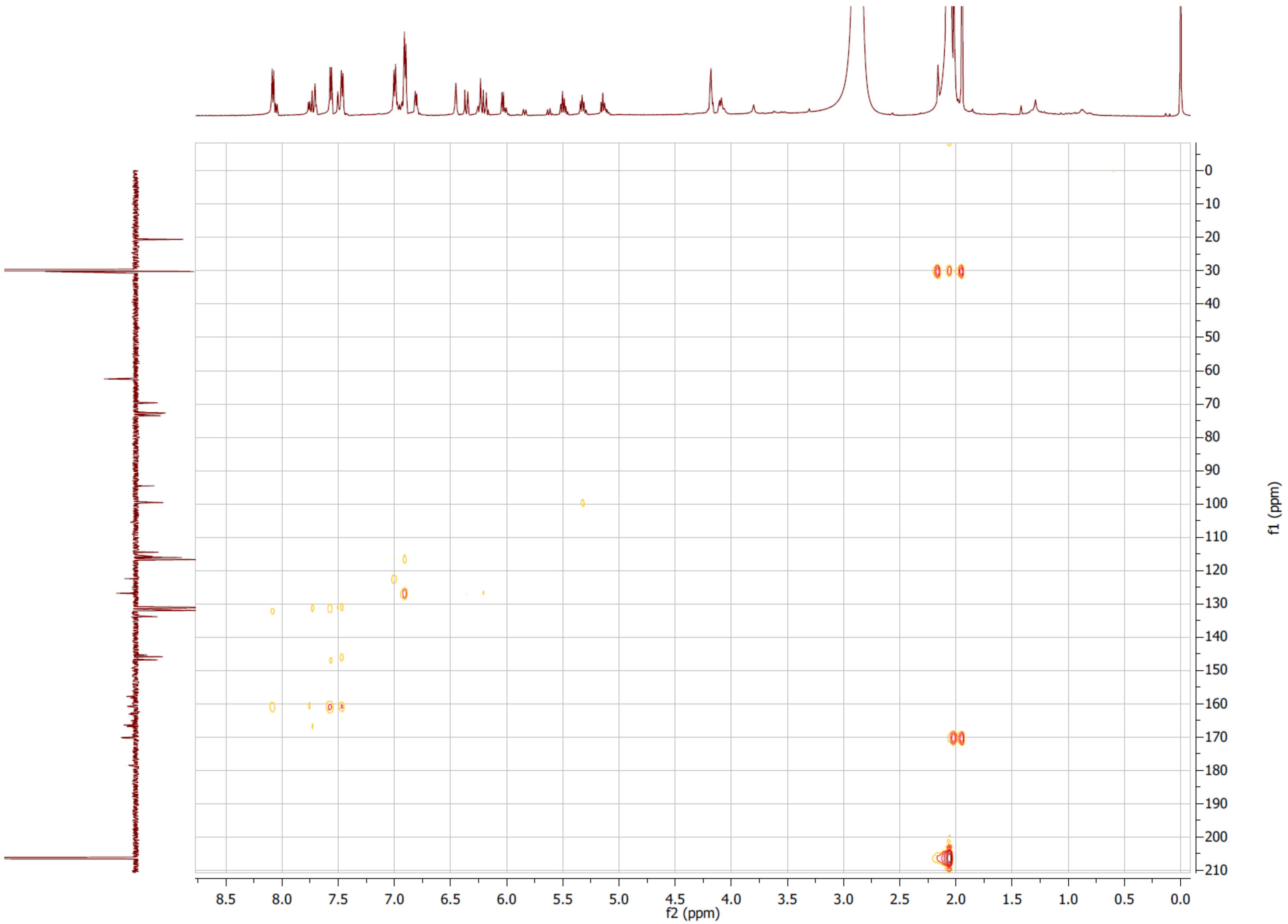
f2 (ppm)
f1 (ppm)
8.5
8.0
7.5
7.0
6.5
6.0
5.5
5.0
4.5
4.0
3.5
3.0
2.5
2.0
1.5
1.0
0.5
0.0
0
10
20
30
40
50
60
70
80
90
100
110
120
130
140
150
160
170
180
190
200
210

Figure S2f

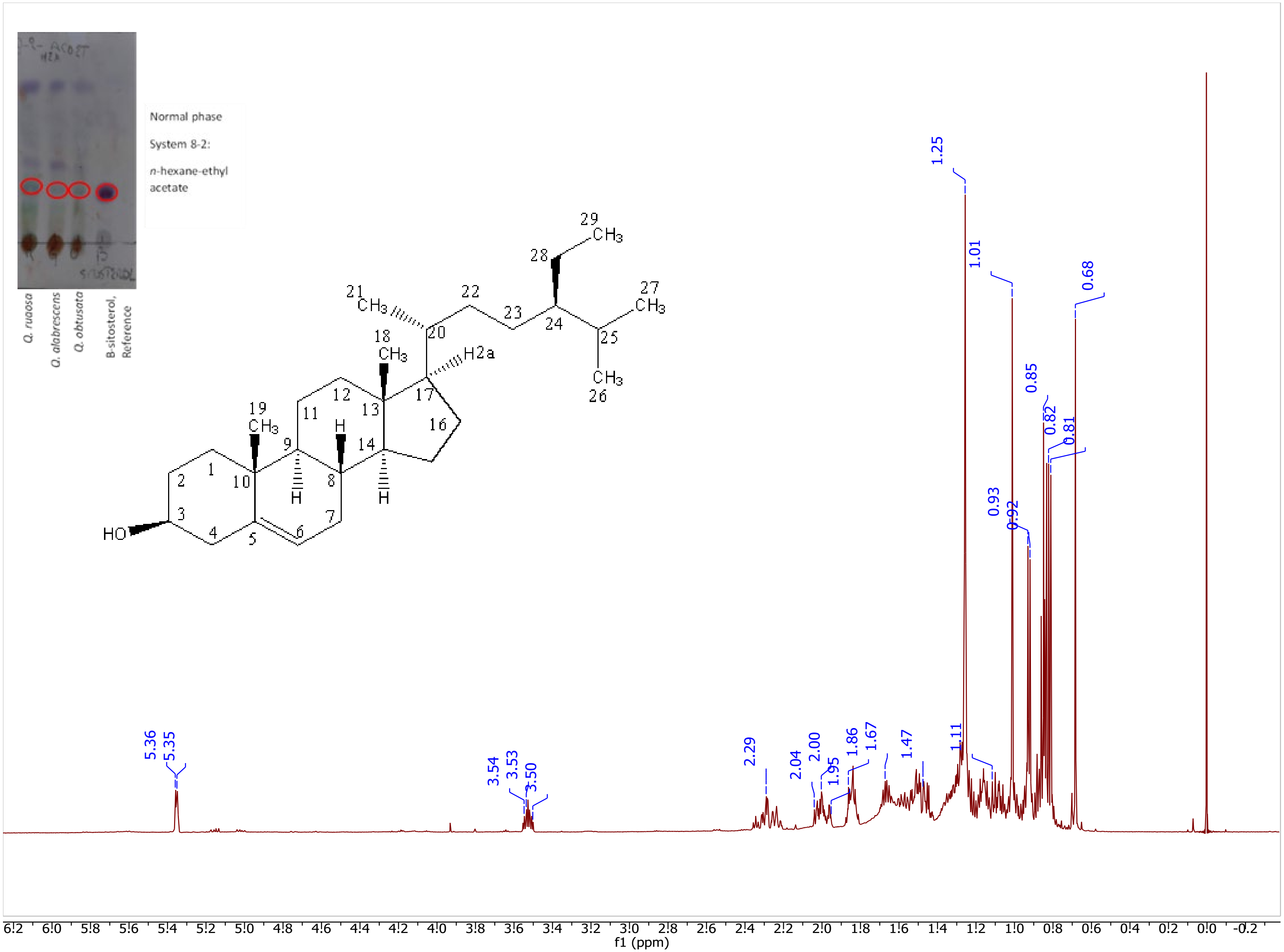

Normal phase
System 8-2:
n-hexane-ethyl acetate
Q. rugosa
Q. glabrescens
Q. obtusata
B-sitosterol, Reference
HO
f1 (ppm)

Figure S3a

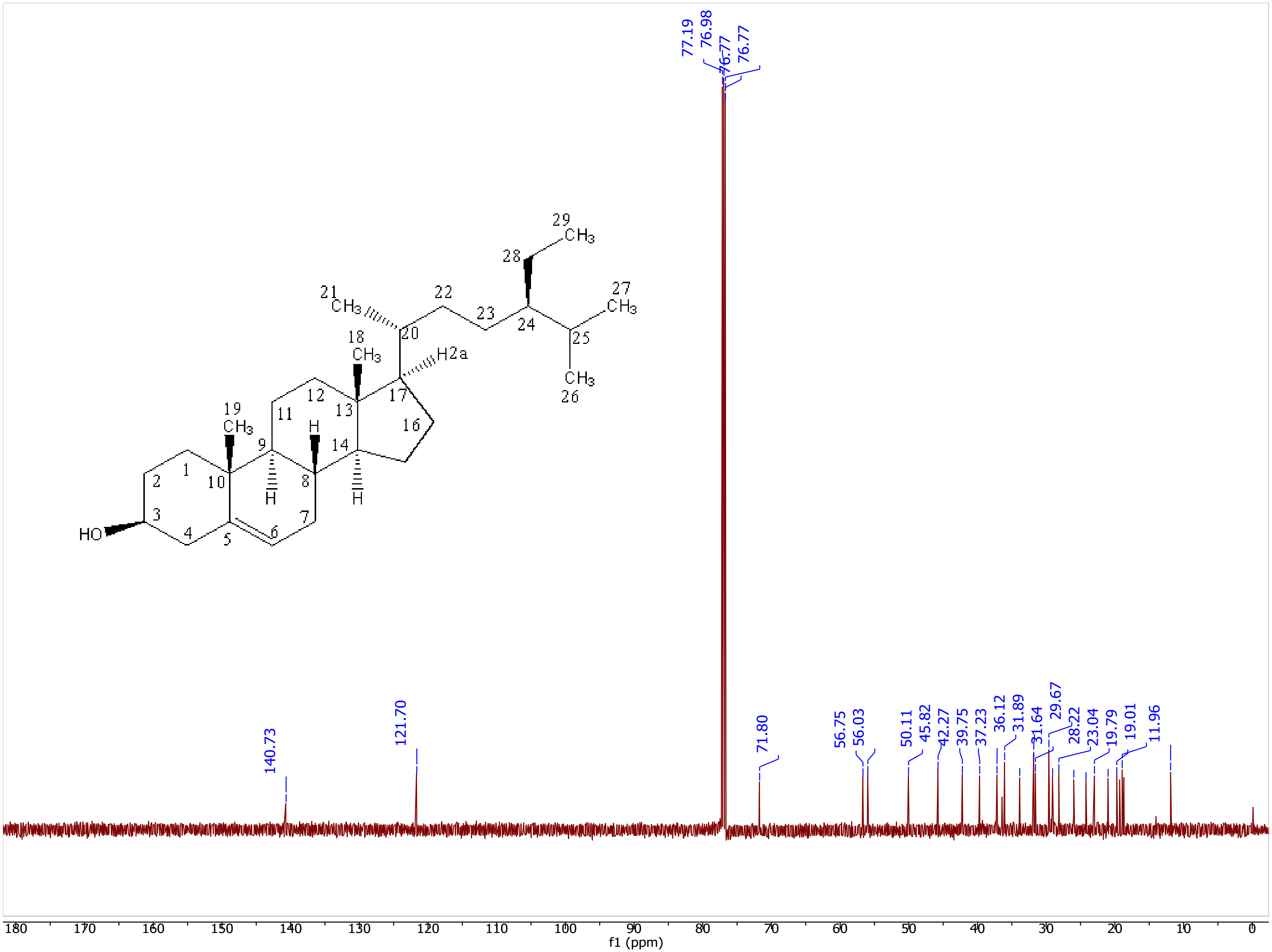

HO
CH3
19
18
21
22
23
24
25
26
27
28
29
H2a
H
1
2
3
4
5
6
7
8
9
10
11
12
13
14
16
17
20
77.19
76.98
76.77
76.77
140.73
121.70
71.80
56.75
56.03
50.11
45.82
42.27
39.75
37.23
36.12
31.89
31.64
29.67
28.22
23.04
19.79
19.01
11.96
180
170
160
150
140
130
120
110
100
90
80
70
60
50
40
30
20
10
0
f1 (ppm)

Figure S3b

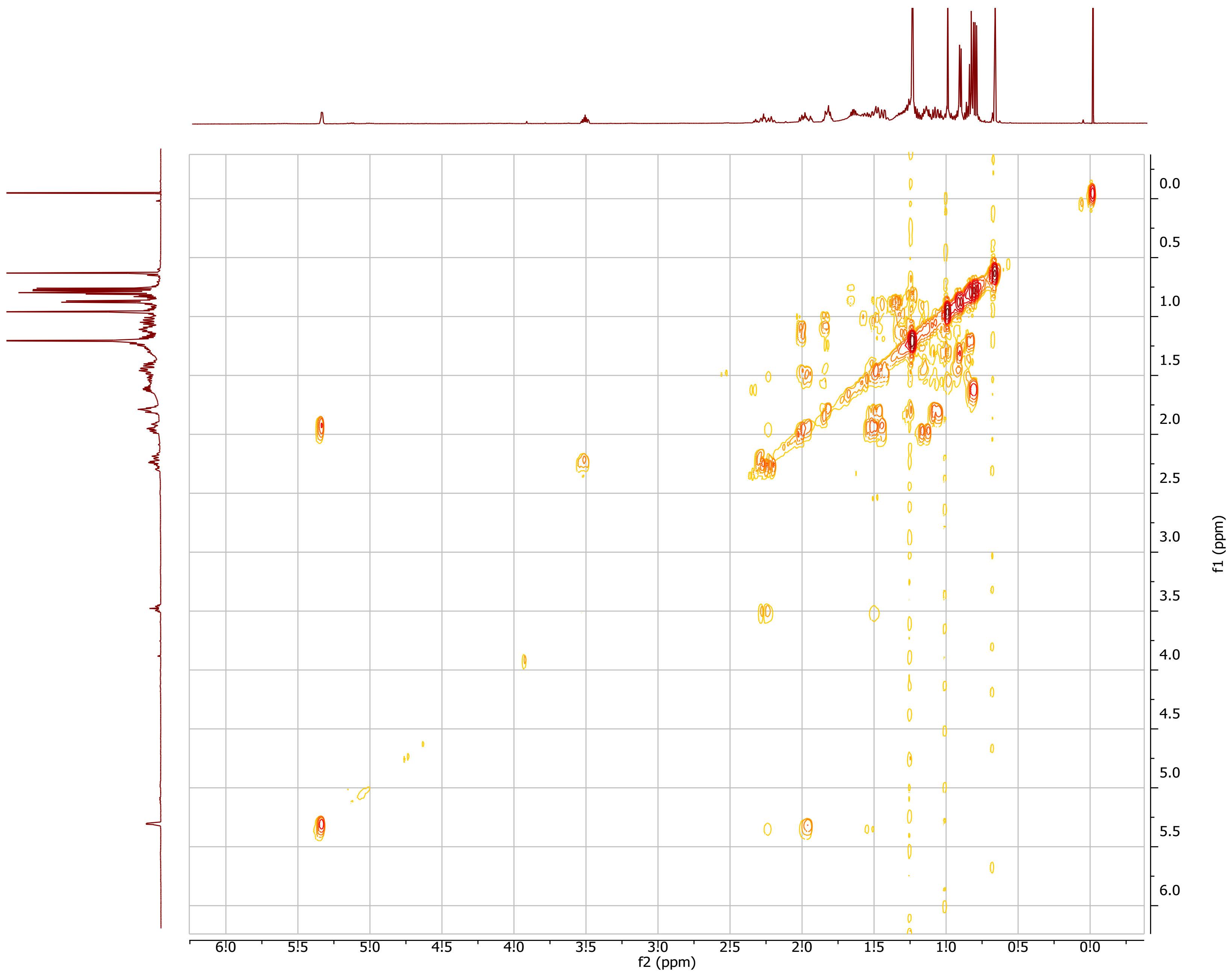

Figure S3c

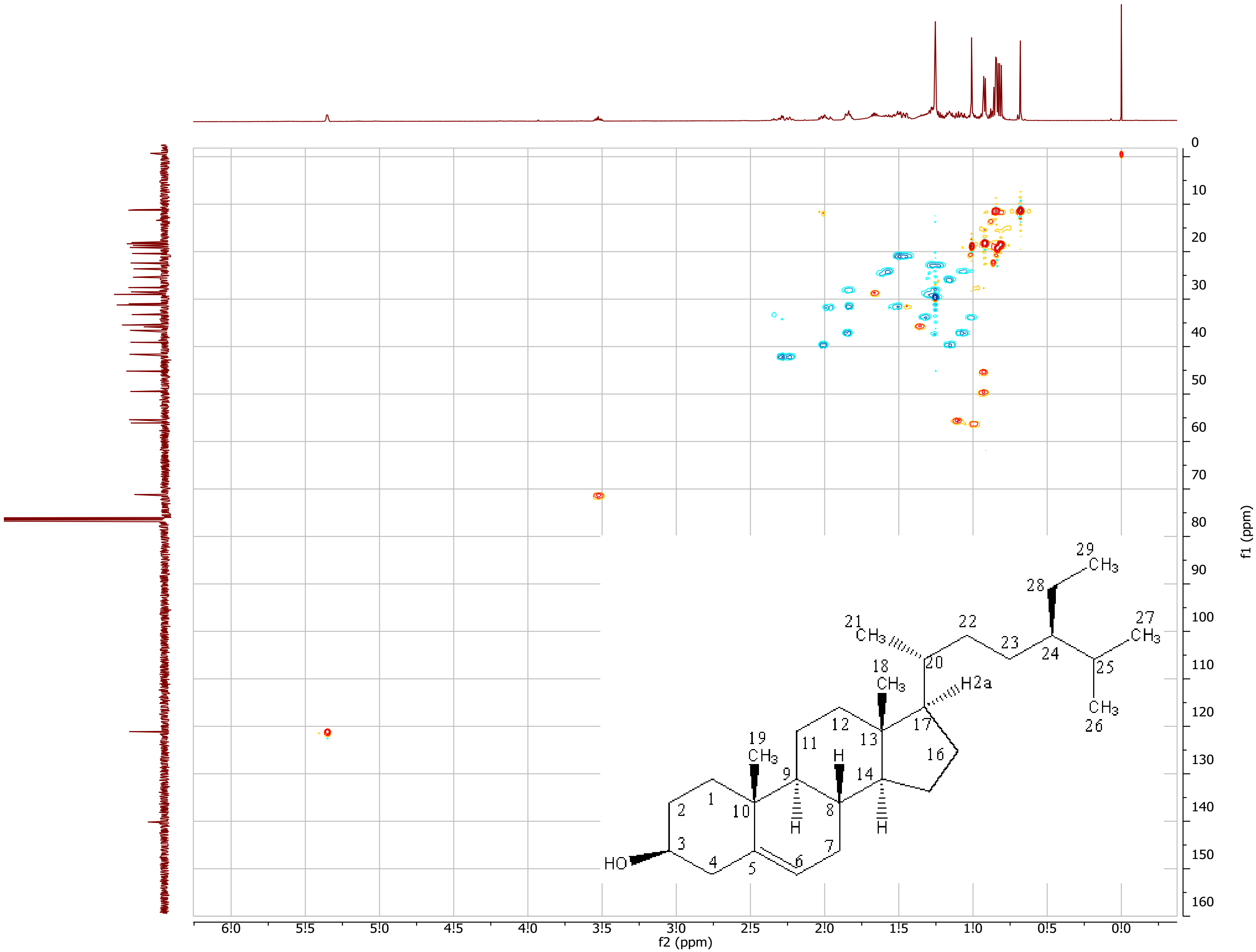
f2 (ppm)
f1 (ppm)
6.0 5.5 5.0 4.5 4.0 3.5 3.0 2.5 2.0 1.5 1.0 0.5 0.0
0 10 20 30 40 50 60 70 80 90 100 110 120 130 140 150 160
HO
19 CH3
18 CH3
21 CH3
29 CH3
27 CH3
CH3 26
H2a

Figure S3d

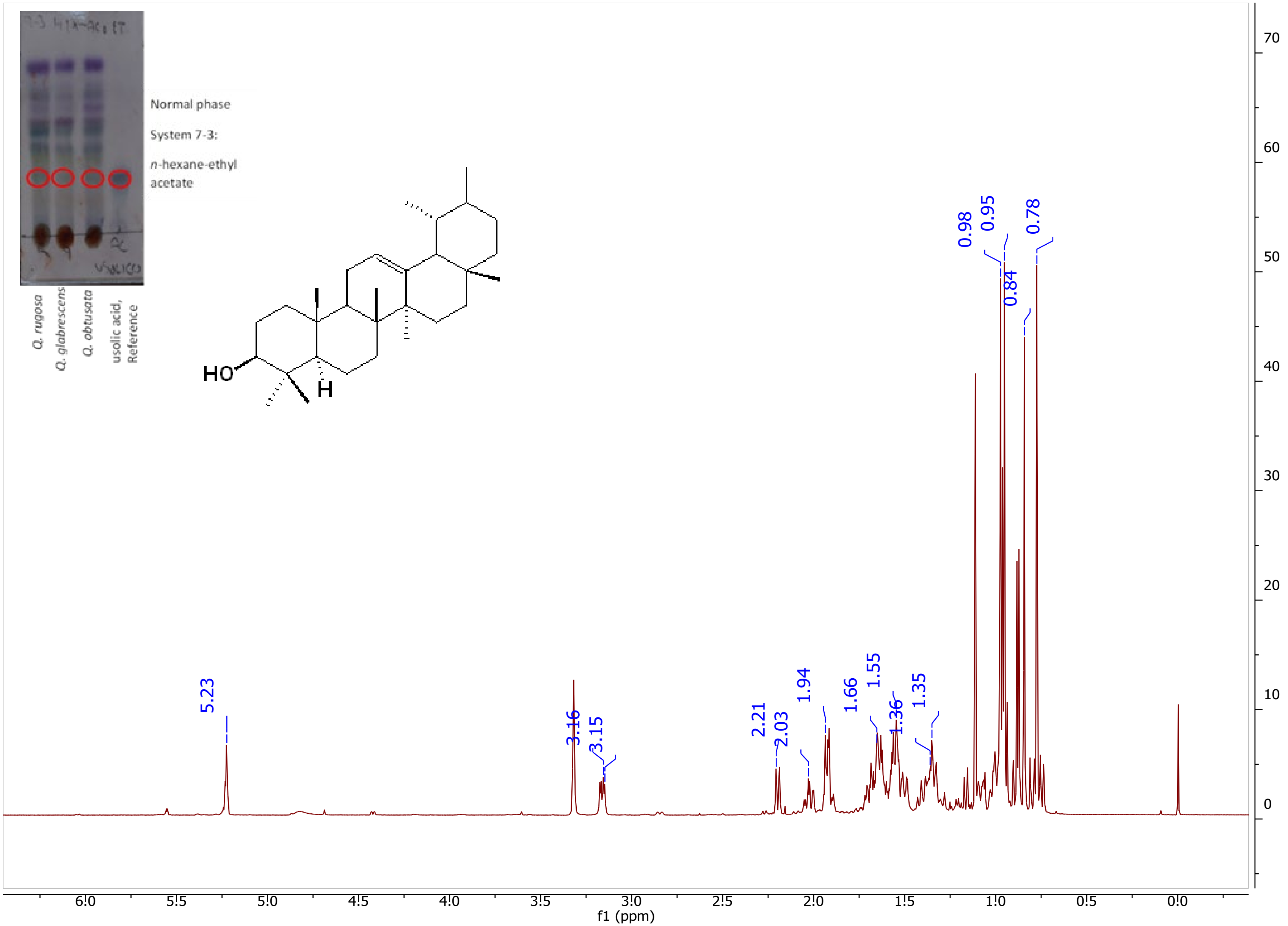
Normal phase
System 7-3:
n-hexane-ethyl acetate
Q. rugosa
Q. glabrescens
Q. obtusata
usolic acid, Reference
HO
H
5.23
3.16
3.15
2.21
2.03
1.94
1.66
1.55
1.36
1.35
0.98
0.95
0.84
0.78
f1 (ppm)

Figure S4

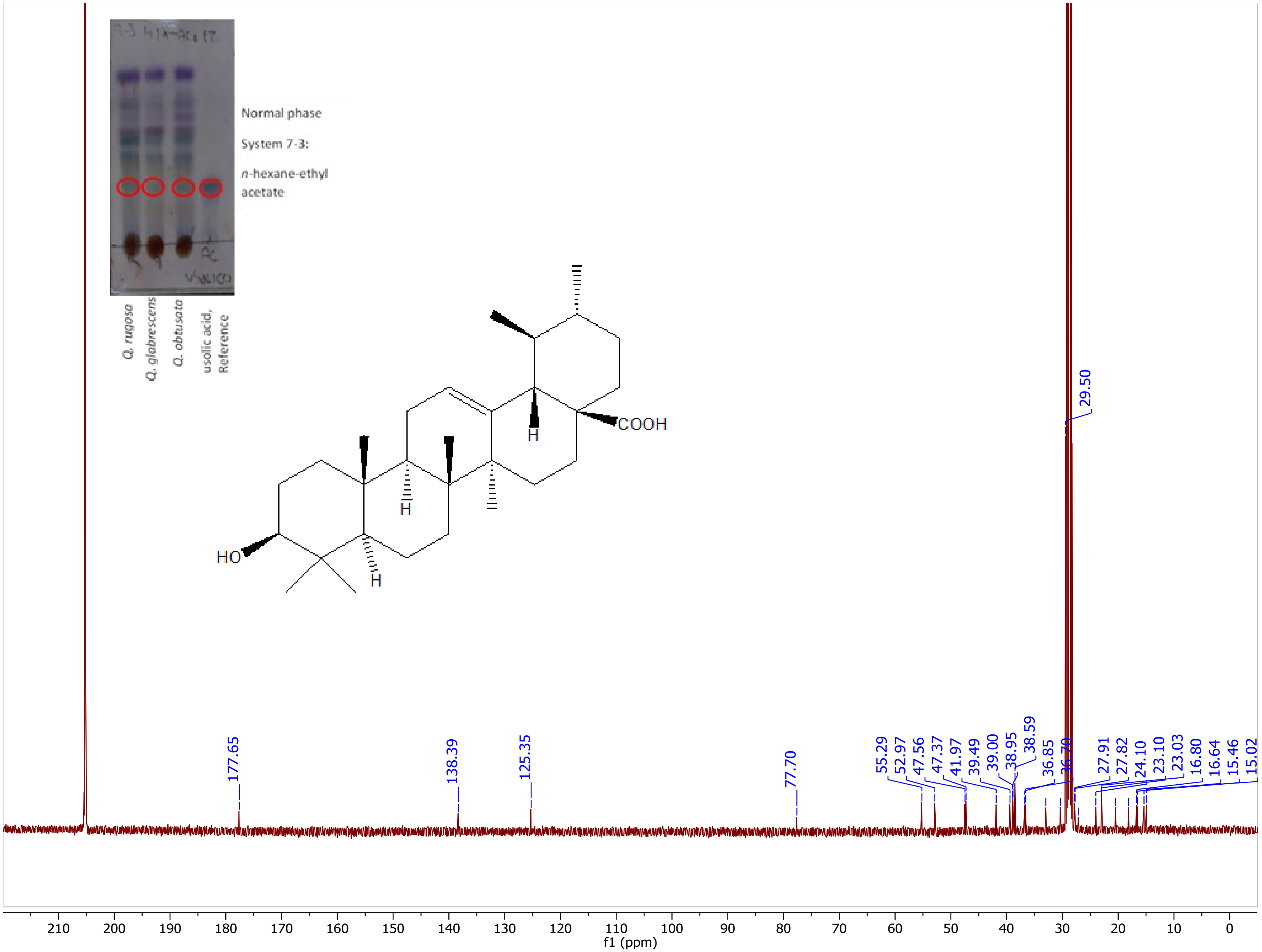
Normal phase
System 7-3:
n-hexane-ethyl acetate
Q. rugosa
Q. glabrescens
Q. obtusata
usolic acid, Reference
HO
H
COOH
177.65
138.39
125.35
77.70
55.29
52.97
47.56
47.37
41.97
39.49
39.00
38.95
38.59
36.85
29.50
27.91
27.82
24.10
23.10
23.03
16.80
16.64
15.46
15.02
f1 (ppm)

Figure S5
